# HSF-1: Guardian of the Proteome Through Integration of Longevity Signals to the Proteostatic Network

**DOI:** 10.3389/fragi.2022.861686

**Published:** 2022-07-08

**Authors:** Maria I. Lazaro-Pena, Zachary C. Ward, Sifan Yang, Alexandra Strohm, Alyssa K. Merrill, Celia A. Soto, Andrew V. Samuelson

**Affiliations:** ^1^ Department of Biomedical Genetics, University of Rochester Medical Center, Rochester, NY, United States; ^2^ Department of Biology, University of Rochester, Rochester, NY, United States; ^3^ Department of Environmental Medicine, University of Rochester Medical Center, Rochester, NY, United States; ^4^ Toxicology Training Program, University of Rochester Medical Center, Rochester, NY, United States; ^5^ Department of Pathology, University of Rochester Medical Center, Rochester, NY, United States; ^6^ Cell Biology of Disease Graduate Program, University of Rochester Medical Center, Rochester, NY, United States

**Keywords:** aging, proteostasis, cell stress and aging, HSF-1 = heat-shock factor-1, longevity, *Caenorhabditis elegans* (*C. elegans*), genetics

## Abstract

Discoveries made in the nematode *Caenorhabditis elegans* revealed that aging is under genetic control. Since these transformative initial studies, *C. elegans* has become a premier model system for aging research. Critically, the genes, pathways, and processes that have fundamental roles in organismal aging are deeply conserved throughout evolution. This conservation has led to a wealth of knowledge regarding both the processes that influence aging and the identification of molecular and cellular hallmarks that play a causative role in the physiological decline of organisms. One key feature of age-associated decline is the failure of mechanisms that maintain proper function of the proteome (proteostasis). Here we highlight components of the proteostatic network that act to maintain the proteome and how this network integrates into major longevity signaling pathways. We focus in depth on the heat shock transcription factor 1 (HSF1), the central regulator of gene expression for proteins that maintain the cytosolic and nuclear proteomes, and a key effector of longevity signals.

## Overview of the Genetics of Longevity in *C. elegans*

### Regulation of Insulin-Like Signaling Determines Metazoan Aging

Perhaps the most profound discovery in the last 30 years of aging research was the discovery in *Caenorhabditis elegans* that aging is under genetic control. Single mutations found in *age-1* and *daf-2* were the first genetic evidence that longevity is influenced by regulated processes (Friedman and Johnson 1988; Johnson 1990; Kenyon et al., 1993). Shortly after this discovery, genetic and molecular analysis revealed that *age-*1 *and daf-*2 function as a part of a conserved insulin-like signaling (ILS) pathway (Dorman et al., 1995). A seminal discovery in *C. elegans* was that the ILS promotes aging by repressing the FOXO transcription factor DAF-16 in wild-type animals (Lin et al., 1997; Ogg et al., 1997). This had major implications for the understanding of diabetes and cancer as it linked a specific transcription factor to mammalian insulin and insulin growth factor (IGF-1) for the first time [for a synopsis of this period of discovery see (Kenyon 2011)]. A large body of work has since demonstrated that this core signaling pathway influences aging across metazoan animals (Holzenberger et al., 2003; Kenyon 2005; Suh et al., 2008).

Early discoveries in the genetics of longevity using *C. elegans* were founded on prior genetic insight into dauer formation (Riddle et al., 1981). Developing *C. elegans* proceed through for larval stages (L1–L4), marked by molts before becoming reproductive adults (Cassada and Russell 1975; Felix and Duveau 2012; Frezal and Felix 2015; Lazetic and Fay 2017). At the L2 to L3 transition, *C. elegans* can enter an alternative L3 developmental stage called dauer, which is a genetic program of developmental diapause, allowing animals to survive food scarcity, overcrowding, or a number of harsh environmental conditions and survive for up to 4 months (Golden and Riddle 1984a; Butcher et al., 2007; Fielenbach and Antebi 2008; Felix and Duveau 2012). Genetic epistasis between mutations that favored constitutive dauer formation under normal conditions, with mutations that rendered animals unable to enter dauer (dauer defective), delineated not only the pathways that regulated dauer development but also informed early subsequent discoveries into the genetics of longevity, which occurred prior to knowing the molecular identity of pathway components (Vowels and Thomas 1992; Kenyon et al., 1993; Gottlieb and Ruvkun 1994; Dorman et al., 1995; Larsen et al., 1995; Gems et al., 1998; Fielenbach and Antebi 2008). It is worth noting that the mechanisms regulating dauer entry and longevity are separable; for example, *C. elegans* with temperature sensitive *daf-2* mutations grown at the permissive temperature bypass the dauer checkpoint and develop into normal adulthood, shifting to higher non-permissive temperatures reduces ILS and extends longevity in these adult animals (Golden and Riddle 1984b; Ailion and Thomas 2000; Ailion and Thomas 2003). This genetic analysis revealed two parallel genetic pathways that converge to regulate dauer development, which we now know are the ILS and TGF-β signaling pathways (Ren et al., 1996; Kimura et al., 1997).

Early studies to identify the Daf genes revealed that organismal longevity was determined via canonical ILS signaling. DAF-2 (insulin/IGF1 receptor) activates AGE-1 (PI3K) to generate phosphatidylinositol-3,4,5-triphosphate (PIP_3_) (Morris et al., 1996; Kimura et al., 1997), which is opposed by DAF-18 (PTEN, Phosphatase And Tensin Homolog) (Ogg and Ruvkun 1998). Accumulation of PIP_3_ activates PDK-1 (PDPK1, 3-phosphoinositide-dependent protein kinase-1), which activates AKT-1/2 (AKT Serine/Threonine Kinase, protein kinase B) via phosphorylation (Paradis et al., 1999). In turn activated AKT-1/2 directly inhibits DAF-16 (FOXO) through phosphorylation and sequestration in the cytosol (Paradis and Ruvkun 1998; Lee et al., 2001; Lin et al., 2001). Mutations within this pathway that decreased ILS lead to activation of DAF-16 and increased longevity (Kenyon et al., 1993; Lin et al., 1997; Ogg et al., 1997). Conversely, mutations that promote ILS, such as loss of *daf-18*, decrease lifespan (Larsen et al., 1995; Ogg and Ruvkun 1998).

Early genetic evidence suggested that TGF-β signaling regulated dauer formation but not lifespan (Larsen et al., 1995). However, it was later discovered that the longevity-regulating activity of the TGF-β pathway was masked by an egg-laying (Egl) phenotype that caused mortality from internal hatching of progeny; suppressing the latter by preventing production of progeny revealed that mutations within the TGF-β pathway doubled lifespan and induced transcriptional changes that overlapped with many DAF-16 regulated genes (Shaw et al., 2007). Molecular genetic analysis of Daf genes revealed an endocrine network that converges within steroidogenic tissues to promote production of a cholesterol derived ligand (dafachronic acid) of DAF-12, which encodes a nuclear hormone receptor orthologous to vertebrate farnesoid-X, liver-X and vitamin D-receptors [reviewed in (Antebi 2013)]. Significantly, many of the endocrine pathways that regulate dauer and longevity are evolutionarily conserved (Mooijaart et al., 2007; Keisala et al., 2009; Kenyon 2010; Tennessen and Thummel 2011).

One unresolved question is the *insulin paradox* in humans. Defects in insulin receptor signaling causes insulin resistance and diabetes. Deficiencies in IGF-1 or upstream growth hormone (GH) are associated with increased incidence of cardiovascular disease and atherosclerosis. Yet, polymorphisms in many components of insulin or IGF-1 pathways that decrease signaling is associated with improved longevity and found in centenarian populations (IGF-1R, PI3K, INSR, FOXO3). Furthermore, centenarian populations are associated with improved insulin sensitivity, low-serum IGF-1, and a mutation in the insulin receptor has been found in semi-supercentenarians (>105 years) [reviewed in (Arai et al., 2009; Calvo-Ochoa and Arias 2015)]. Early characterization of *C. elegans* with difference alleles of *daf-2,* all of which increase lifespan, noted different degrees of an effect on motility, stress resistance, morphology, development, reproductive lifespan and brood size (Gems et al., 1998). Mutations within the ligand binding domain tended to have less pleiotropies in contrast to mutations within the kinase domain of DAF-2, which suggested DAF-2 kinase activity has at least separable outputs on organismal physiology (i.e. longevity and other pleotropic effects) (Gems et al., 1998). Whether different alleles of *daf-2* differentially impact the molecular and cellular hallmarks of aging (discussed below) remain unexplored.

One possible explanation of the insulin paradox is that there is an optimal reduction in ILS to increase longevity, and reduction below this rate results in metabolic syndromes and premature aging (Cohen and Dillin 2008). Consistent with that possibility, null-mutations of *daf-2* and *age-1* in *C. elegans* result in lethal constitutive dauer formation (Malone and Thomas 1994; Dorman et al., 1995; Larsen et al., 1995; Gems et al., 1998) and several *daf-2* mutations are temperature sensitive loss of function: small increases in lifespan are observed at lower permissive temperatures and greater increases when temperature is increased (Gems et al., 1998). However, *age-1* null mutant animals raised at lower temperature can bypass dauer and eventually develop into adults that have near normal feeding rates, motility, and remarkably live up to 145–190 days, which is 10-times longer than wild-type animals (Ayyadevara et al., 2008). This suggests that the insulin paradox cannot be solved based simply on levels of ILS. Possible explanations to the insulin paradox that are not mutually exclusive include: differences between insulin and IGF-1 signaling, tissue- or cell-type specific effects, background mutations, the nature of mutation, and timing of alterations in ILS.

### An Emerging Theme: Genes and Pathways Linked to Metabolic Control Determine Aging

Since the discovery that ILS regulates longevity, a common theme has emerged: the evolutionarily conserved genes and pathways that have the largest impact on lifespan often act in nutrient and energy sensing. For instance, a key controller of nutrient sensing is the target of Rapamycin (TOR) response to decreased levels of amino acids and carbohydrates (Kapahi et al., 2010). Under nutrient-rich conditions, TOR promotes cellular growth by simultaneously activating protein translation (*e.g.,* transcription of translation components) while inhibiting protein turnover (*e.g.,* transcription of chaperone and autophagy genes (McCormick et al., 2011; Seo et al., 2013; Lapierre et al., 2015)), and by inhibiting the initiation of autophagy. TOR inhibition, or activation of targets of TOR inhibition, results in extension of longevity (Jia et al., 2004; Hansen et al., 2008). Similarly, AMP-activated protein kinase (AMPK) acts as a conserved energy sensor of increased levels of AMP and ADP. Energy-stress activation of AMPK induces autophagy, the oxidative stress response (OSR), and extends longevity (Apfeld et al., 2004; Greer et al., 2007; Greer et al., 2009; Salminen and Kaarniranta 2012). Sirtuins (SIRT1-7 in mammals, *sir-2.1* and *sir-2.4* in *C. elegans*) also play a key role in nutrient sensing and extension of longevity (Jedrusik-Bode et al., 2013; Jedrusik-Bode 2014). Sirtuins are (NAD+)-dependent deacetylases, which sense levels of NAD+, an important metabolite linked to longevity (Verdin 2015). Sirtuin-mediated extension of longevity has been linked to ILS, AMPK, and TOR signaling, and sirtuins are essential for both dietary restriction (DR) and exercise to increase lifespan (Dai et al., 2018). From these and many additional studies two conclusions become self-evident: aging is under genetic control and these mechanisms have been deeply conserved throughout evolution.

Why would nutrient sensing, the abundance of key metabolites and energy currency, be causally linked to genetic programs that determine organismal longevity? It is tempting to speculate that very early in evolution organisms able to couple physiology to energy resources had a survival advantage. Under conditions of plentiful resources, organisms able to develop and reproduce quickly could dominate an ecological niche. However, when food is scarce, organisms able to conserve or recycle resources and delay energetically-costly physiological processes. such as development and reproduction, in favor of mechanisms that protect the Soma, would have a survival advantage. Delaying the production of offspring has the added benefit of limiting competition for limited resources. Consistent with this hypothesis, in *C. elegans* many long-lived mutant animals have one or more of the following characteristics: slower development, links to dauer formation (a form of developmental diapause), reduced numbers of overall progeny, and/or an extended period of progeny production (i.e., reproductive span) (Szewczyk et al., 2006; Mukhopadhyay and Tissenbaum 2007). In fact, loss of the *C. elegans* germline through mutation of *glp-1*, which encodes an ortholog to the Notch receptor, increases lifespan by preventing germ cell development in early adulthood, which also requires DAF-16, implying a connection to ILS (Arantes-Oliveira et al., 2002).

Refined genetic analysis has revealed that many of the phenotypes associated with reproduction or development are separable from longevity. For example, early discoveries in *C. elegans* aging research using temperature-sensitive alleles in the ILS pathway revealed that dauer formation and extended longevity were genetically separable (Kenyon et al., 1993), implying that strategies to improve healthy aging based on the genetics of longevity may not require a cost in developmental or reproductive fitness.

The aforementioned longevity signals converge on a limited number of transcription factors, which also respond to numerous additional stress signals. For example, *skn-1* encodes the *C. elegans* ortholog of the nuclear factor erythroid 2-related factor 2 (Nrf2), a member of the “Cap’n'Collar” basic leucine zipper family of transcription factors, which is best known for regulating the expression of the OSR (An and Blackwell 2003). However, specific splice isoforms of *skn-1* play key roles in the endoplasmic reticulum (ER) unfolded protein response (ER-UPR), maintaining proteostasis in the cytosol, and the response to starvation (Glover-Cutter et al., 2013; Lehrbach and Ruvkun 2016; Denzel et al., 2019; Lehrbach and Ruvkun 2019). DR activates SKN-1 within two head neurons (ASI) and is essential for increased longevity and cell non-autonomous changes in metabolic activity within peripheral tissues (Bishop and Guarente 2007). Additionally, amino acid and carbohydrate starvation activate *skn-1* through TOR signaling (Robida-Stubbs et al., 2012), and reduced ILS activates SKN-1 in conjunction with DAF-16 (Tullet et al., 2008). Additional evidence of signal convergence is AMPK phosphorylation and activation of DAF-16, after a distinct method of DR (Greer et al., 2007; Greer et al., 2009). Furthermore *pha-4,* which encodes the FOXA forkhead transcription factor is critical for lifespan extension phenotypes related to germline inhibition and DR, but not reduced ILS, through regulation of autophagy (Panowski et al., 2007; Lapierre et al., 2011). One of the key transcriptional effectors of longevity signaling is the heat shock transcription factor (HSF-1 in *C. elegans*, HSF1 in more complex metazoans), which we discuss in detail in the latter part of this review. Collectively, a growing number of *C. elegans* studies have begun to unravel the complex integrated networks that maintain organismal homeostasis from an extensive array of diverse extrinsic and intrinsic signals that converge on distinct but overlapping adaptive transcriptional responses (Greer and Brunet 2009; Denzel et al., 2019).

### Longevity is Determined *via* Cell Non-Autonomous Signals

A strength of *C. elegans* as a model is the relative ease in achieving tissue- and cell-type specific genetic perturbation. Overexpression or rescue is easily achieved through the use of either tissue specific promoters (in wild-type or mutant backgrounds) or through mosaic analysis (Yochem et al., 2000; Yochem and Herman 2003; Meister et al., 2010; Prelich 2012). Spatial and temporal gene inactivation can be achieved classically through the use of tissue specific RNAi (e.g., tissue specific expression of *rde-1* in RNAi-deficient *rde-1* mutant animals) (Qadota et al., 2007; Miles and van Oosten-Hawle 2020; Watts et al., 2020) or more recently with the development of the Tir1-auxin system, which provides spatial and temporally controlled protein degradation (Zhang et al., 2015). Collectively, these approaches allowed early efforts in the emerging field of aging research identify the tissues where a longevity gene or pathway functioned. These studies raised two possibilities: 1) longevity functions restricted to a specific tissue are regions with a metazoan that decline cell-intrinsically during aging and ultimately result in death, or 2) the longevity functions within a specific tissue generate cell non-autonomous paracrine or endocrine signals that orchestrate cellular aging across tissues. The latter view has become widely accepted and is based on numerous studies ranging from early observations to ongoing discoveries. Distinct tissues which influence aging throughout the Soma through endocrine signals include neurons, the somatic gonad, the germ line/gonadal stem cells, and intestinal cells (Kleemann and Murphy 2009). Examples of some of the longevity signaling pathways that act cell non-autonomously through endocrine signaling include ILS, bile acid signaling, TGF-β signaling, serotonin signaling, pregnenolone signaling, TORC1 signaling, and AMPK signaling (Kleemann and Murphy 2009; Ulgherait et al., 2014; Zhang et al., 2019). Signaling is likely to occur through lipophilic hormones (Hansen et al., 2005). These signals are distinct, for example: alterations in gonadal stem cell signaling communicates cell non-autonomously with somatic intestinal cells through lipophilic hormone signaling and *kri-*1 (ortholog of human KRIT1) to activate DAF-16. This cell non-autonomous mechanism is specific to germline to Soma signaling, as these functions are not required for ILS-mediated longevity or DAF-16 activation (Berman and Kenyon 2006). Similarly, endocrine germline signals and DR differentially regulate protein quality control mechanisms (Shpigel et al., 2019). For a recent review on cell non-autonomous signaling in longevity see (Miller et al., 2020).

### Hallmarks of Molecular and Cellular Aging

Many molecular and cellular hallmarks of aging have been discovered, which have been broadly classified as: altered intercellular communication, genomic instability, telomere attrition, epigenomic alterations, deregulated nutrient sensing, mitochondrial dysfunction, cellular senescence, stem cell exhaustion and dysfunction, and decline of protein homeostasis (proteostasis) (Lopez-Otin et al., 2013). While many of the mechanisms that alter aging impact multiple hallmarks, and similarly these hallmarks impact one another; in this review we focus primarily on proteostasis through an in depth discussion of the mechanisms that preserve proper function and folding of the cytosolic and nuclear proteomes, how these mechanisms intersect with the aforementioned longevity signals, and give special emphasis to the key transcription factor that acts as the guardian of the nuclear and cytosolic proteome: HSF1. We provide a detailed analysis of discoveries in mammals and *C. elegans,* and highlight areas of future investigation where iterative analysis between systems would provide deeper mechanistic insight to how proteostasis preserves the functional integrity of complex metazoans.

## Inherent Challenges to Maintaining Proteostasis

A challenge inherent to proteostasis is ensuring all proteins are properly folded *de novo* into a native conformation and maintained in a soluble state, despite overall protein concentrations approaching levels found in crystals (Powers et al., 2009). This is further amplified by the varied nature of protein size, amino acid composition, structural conformation, stability, turnover, and expression (Wolff et al., 2014). Thus, maintaining proteostasis is a generalized problem, not unique to a subset of proteins. Layered within this complexity is the need for proteins to be localized within the proper cellular compartment, to be maintained in correct stoichiometric ratios relative to other components of larger protein complexes, to undergo the correct modifications in response to a diverse array of internal and extrinsic cues, and to be maintained within a proteome with a composition unique to each cell type or tissue. To put it simply, the proper function and folding of the proteome is a complex and dynamic process, vital for maintaining cellular function.

## Overview of the Proteostatic Network

**FIGURE 1:**
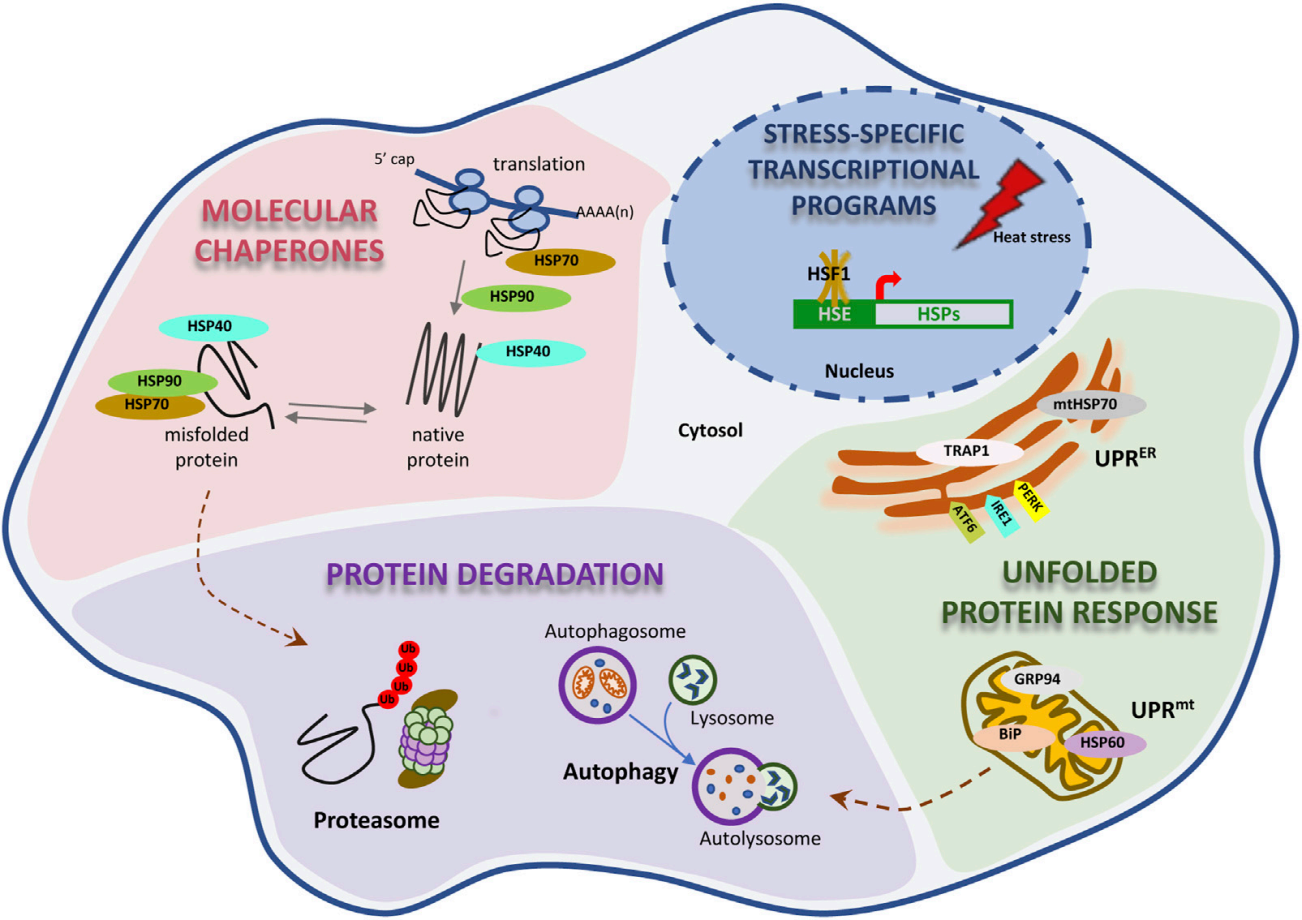
Proteostasis is maintain by a proteostasis network. Proteostasis has multiple levels of regulation. A great number of molecular chaperones are present in the cytosol, assisting on the appropriate folding and quality of the proteome. The proteosome and autophagy protein degradation pathways are essential for the clearance of unneeded proteins. Two distinct, but analogous, unfolded protein responses in the mitochondria and endoplasmic reticulum (mitoUPR and ER-UPR, respectively) are necessary to activate the expression of chaperones and to protect the function of these organelles from unfolding stress. Ultimately, the PN network is equipped with the adaptive activation of different transcriptional programs that get induced by different types of stress.

Proteostasis is maintained through the coordinated action of a large proteostatic network (PN), consisting of approximately 2,000 unique proteins, which regulate the synthesis, folding, trafficking, and degradation within the proteome [reviewed in (Morimoto 2008; Wolff et al., 2014; Hipp et al., 2019; Morimoto 2019)]. This network regulates *de novo* protein folding from the emergence of the nascent polypeptide from the ribosome to the final subcellular localization of the mature protein. The PN responds to acute stress on the proteome, and fine-tunes the rates of translation and degradation in response to a myriad of cell intrinsic and extrinsic cues. The major components are several large families of molecular chaperones, co-chaperones, and protein clearance mechanisms, predominately degradation through the ubiquitin-proteasome system (UPS) and autophagy at the lysosome (Figure 1).

## Declining Proteostasis During Aging

### Decline of Cellular Proteostasis in *C. elegans*

Key discoveries in *C. elegans* first revealed that declining proteostasis is a hallmark of aging, and provided a generalized cellular explanation for the manifestation and progression of neurodegenerative disease. The decline of cellular proteostasis is a hallmark of aging across species [reviewed in (Rubinsztein et al., 2011; Taylor and Dillin 2011; Labbadia and Morimoto 2015a; Klaips et al., 2018; Hipp et al., 2019)]*.* For instance, even in the absence of overt disease, there is a growing body of evidence demonstrating that the loss of autophagy and declining levels of molecular chaperones are conserved hallmarks of aging (Ben-Zvi et al., 2009; Morimoto and Cuervo 2009; Vellai et al., 2009). The use of transgenic *C. elegans* expressing polyglutamine fused to a fluorescent reporter remains a powerful system to identify age-associated declines in proteostasis *in vivo* (Satyal et al., 2000; Brignull et al., 2006; Morimoto 2006; Morimoto 2008). Early studies demonstrated that co-expression of both a proteotoxic polyQ reporter and endogenous metastable proteins further exacerbates protein misfolding and degenerative phenotypes, consistent with the notion that prolonged proteotoxic stress overloads the limited buffering capacity of the PN (Gidalevitz et al., 2006). Widespread failures in protein folding have since been shown to occur in early adulthood across tissues and coincides with reduced activation of HSF-1 and chaperone protein expression (Ben-Zvi et al., 2009). In *C. elegans,* the transcriptional inducibility of multiple forms of stress response rapidly decline within a few hours after the onset of reproduction due to the formation of repressive chromatin marks at stress loci (Shemesh et al., 2013; Labbadia and Morimoto 2015a; Labbadia and Morimoto 2015b). During normal aging, solubility of the *C. elegans* proteome declines, resulting in an accumulation of aggregates (David et al., 2010; Reis-Rodrigues et al., 2012; Walther et al., 2015). Additionally, during aging, there is an increase in misfolded and oxidatively-damaged proteins, especially in neurons (Powers et al., 2009; Kundra et al., 2017; Sala et al., 2017).

The age-associated collapse of proteostasis is also intrinsic to human senescence. During aging of higher metazoans, cells can enter a permanent form of G1 arrest commonly referred to as cellular senescence [reviewed in (McHugh and Gil 2018; Prata et al., 2018; Song et al., 2020)]. Cellular senescence is a genetic program (Serrano et al., 1997), and a key hallmark of aging, which can be induced by various age-associated damage drivers including: telomere damage, epigenetic dysregulation, DNA damage, and mitochondrial dysfunction. Accumulating senescence cells play a key role in age-associated diseases by promoting stem cell exhaustion, chronic inflammation, disruption of nutrient signaling, and proteostatic dysfunction (McHugh and Gil 2018; Prata et al., 2018; Borghesan et al., 2020; Song et al., 2020). The age-associated collapse of proteostatic networks observed in *C. elegans* also occurs in human senescent cells. For example, within senescent cells the transcriptional activation of the heat shock response deteriorates, activation of HSF1 is impaired, UPR-related transcriptional responses are impaired, and the proteosome is dysfunctional (Sabath et al., 2020).

## Components of the Proteostatic Network and Links to Longevity

### Molecular Chaperones

The “workhorse” components of the PN are the molecular chaperones, which assist in all aspects of proteostasis both under normal conditions and in response to challenge from intrinsic and extrinsic factors. Chaperones are tightly regulated; their relative abundance is closely linked to the rest of the proteome, with a limited buffering capacity (Gidalevitz et al., 2011). Thus, the consequence of proteotoxic stress is overload upon the chaperone system, resulting in protein misfolding. In the context of proteostatic disease, aggregate formation and toxic gain-of-function disruption of normal cellular physiology occurs and ultimately results in cell death if unresolved (Balch et al., 2008).

As key components of the PN, there are numerous molecular chaperones with diverse regulatory roles in maintaining proper function and folding of the proteome [for excellent reviews see (Richter et al., 2010; Balchin et al., 2016; Hipp et al., 2019)]. An informatic analysis identified 332 human genes encoding chaperones and co-chaperones (Brehme et al., 2014), which fall into nine families: HSP90, HSP70, HSP60, HSP40, prefoldin, small heat shock protein (sHSP), TPR-domain containing (Hartl and Hayer-Hartl 2002), and organellar-specific chaperones of the endoplasmic reticulum (Kleizen and Braakman 2004) and mitochondria (Tatsuta et al., 2005). The number of paralogous genes within a family can be extensive: members may be essential or dispensable for viability, act in specific contexts (e.g., *de novo* protein folding or refolding misfolded proteins), be expressed constitutively or regulated *via* specific signals, be expressed in specific cell types, or be localized in specific subcellular regions (Richter et al., 2010; Balchin et al., 2016). For example, cytosolic HSP70 paralogs differ in that heat shock cognate protein *HSC70* is constitutively expressed, while in contrast expression *HSP70* expression is induced after heat shock. The HSP90s and HSP70s are highly abundant chaperones in the cytosol and nucleus (Taipale et al., 2010; Labbadia and Morimoto 2015a). Paralogs, such as BiP and GRP94 are essential for ER function, while the paralogs mortalin and TRAP1 function in the mitochondria. HSP90s, HSP70s, HSP60s, and sHSPs mediate *de novo* protein folding of nascent polypeptides, a process directly coupled to translation, acting alone or in conjunction with co-chaperones. In response to stress, other HSP70 and HSP90 family members are induced to resolve protein misfolding. Small molecular chaperones are somewhat distinct, as they interact reversibly with a broad range of unfolded substrates independent of ATP. sHSPs do not primarily refold proteins, rather they prevent the formation of highly stable, proteotoxic aggregates, acting as a storage depot for unfolded proteins until they can be refolded or degraded [for detailed reviews on the chaperone system see (Bar-Lavan et al., 2016; Biebl and Buchner 2019; Rosenzweig et al., 2019; Jayaraj et al., 2020; Reinle et al., 2022)].

Molecular chaperones are central to the proper regulation of protein homeostasis in aging cells (Labbadia and Morimoto 2015a; Margulis et al., 2020). Induction of molecular chaperones in response to stress is essential for normal development, and is significantly reduced during aging (Lopez-Otin et al., 2013; Dues et al., 2016; Sabath et al., 2020). In addition to regulating proteostasis, molecular chaperones and co-chaperones have been implicated in *C. elegans* longevity (Hsu et al., 2003; Morley and Morimoto 2004); direct manipulation of chaperone levels can alter *C. elegans* lifespan. For example, loss of HSP90 shortens lifespan (Somogyvari et al., 2018), while overexpression of HSP16 or HSP70 increases lifespan (Lithgow et al., 1995; Tatar et al., 1997; Yokoyama et al., 2002; Walker and Lithgow 2003). Molecular chaperone levels of expression are also regulated by longevity pathways. For instance, ILS regulates molecular chaperone expression, as the long-lived *daf-2* and *age-1* mutant animals have increased expression levels (Hsu et al., 2003; McElwee et al., 2003; Murphy et al., 2003; Walker and Lithgow 2003; Morley and Morimoto 2004; Halaschek-Wiener et al., 2005; Lamitina and Strange 2005; Wentz et al., 2018). Similarly, inactivation of TORC1 (e.g., *daf-15* encodes the *C. elegans* ortholog of Raptor) or downstream S6 kinase (*rsks*-1) is sufficient to induce chaperone expression and interestingly sHSPs, but *hsp70*s are not essential for the increased lifespan of *rsks-1* mutant animals (Seo et al., 2013). Chaperone functions also feedback to longevity signaling pathways. For example, the HSP90 family member DAF-21 directly regulates nuclear localization and transcriptional activity of the DAF-16A isoform (Somogyvari et al., 2018). Chaperones also coordinate adaptive transcriptional responses to changes in longevity signals. For instance, prefoldin 6 (*pfd-6*) encodes a chaperone, which under conditions of decreased ILS integrates HSF-1 and DAF-16 transcriptional activity (Son et al., 2018). These studies highlight a few examples of increasing evidence that molecular chaperones are not merely effectors of mechanisms to maintain the proteome through changes in protein folding, degradation and aggregation. Rather, molecular chaperones play key roles in connecting the PN with longevity signals and pathways.

### Ubiquitin Proteasome System and Autophagy

Protein degradation is a fundamental mechanism for maintaining proteostasis. Turnover of unfolded polypeptides *via* proteasome- and autophagy-mediated degradation pathways are additional components of the PN. These effector mechanisms not only safeguard *de novo* protein quality control but are continually adjusted in response to stress from both internal cues and endocrine signals through transcriptional regulators of the PN.

The UPS is the main protein degradation system within the cell and is an integral part of the PN, assisting by clearing misfolded or toxic proteins. The proteasome is composed by a 19S regulatory cap and a 20S proteolytic core (Finley 2009). The ubiquitinated substrate attaches to the 19S regulatory cap via ubiquitin receptors to be translocated to the 20S core where it is hydrolyzed, effectively degrading it. Substrates targeted to the proteasome are tagged by polyubiquitin chains by a series of steps of E1, E2, and E3 ligases. Targets for degradation are redirected to the proteasome through interaction with co-chaperones, including the C-terminus of HSC70-interacting protein (CHIP) and Bcl2-associated athanogene 1 (BAG1), in conjunction with HSP70 and HSP90 complexes (Connell et al., 2001). When proteome stability cannot be maintained by protein degradation through the UPS, such as after heat shock, the accumulation of misfolded proteins is alleviated through increased autophagy. Thus, the major degradation mechanisms also function as a part of an integrated system.

A second major degradation system is mediated *via* the lysosome. The process of macroautophagy (hereafter referred to as autophagy), chaperone-mediated autophagy, and microautophagy are clearance mechanisms for a growing number of substrates, including proteins, aggregates, damaged organelles, nucleic acids, and pathogens (Rabinowitz and White 2010; Tooze and Yoshimori 2010; Workman and van Montfort 2010; Mizushima 2011; Benbrook and Long 2012; Pyo et al., 2012; Khaminets et al., 2015; Khaminets et al., 2016). Like the ubiquitin-dependent proteasome pathway, autophagy is tightly regulated (He and Klionsky 2009; Kroemer et al., 2010). Autophagosomes, which are double-membrane vesicles, form during autophagy to sequester substrates for degradation. These loaded autophagosomes then fuse to lysosomes to form autolysosomes, and substrates are degraded by lysosomal hydrolytic activity. The activation of autophagy is important to protect cells against multiple stressors such as heat shock and nutrient deprivation (Kroemer et al., 2010), protecting the organism from diseases associated with degeneration, infections, and inflammation, among others (Levine and Kroemer 2008; Mizushima et al., 2008; Aman et al., 2021; Kaushik et al., 2021; Nieto-Torres and Hansen 2021).

The UPS is essential for normal aging in *C. elegans* (Papaevgeniou and Chondrogianni 2016; Margulis et al., 2020; Ottens et al., 2021). The ubiquitin E3 ligase CHIP promotes longevity through ILS by regulating insulin receptor turnover (Tawo et al., 2017). Additionally, the CUL-1 E3 ligase complex regulates DAF-16 transcriptional activity (Ghazi et al., 2007). Another ubiquitin E3 ligase, RLE-1, targets DAF-16 for polyubiquitination-mediated degradation (Li et al., 2007). The proteasome itself also plays a key role in aging. Multiple studies have shown that loss of proteasome subunits lead to premature aging (Yun et al., 2008; Chondrogianni et al., 2015). Upregulated proteasomal activity is observed in the long-lived *glp-1* mutant animals and in DR models (Vilchez et al., 2012; Depuydt et al., 2013). The role of deubiquitination enzymes (DUBs) has also been implied in proteasome activity and aging in *C. elegans* (Papaevgeniou and Chondrogianni 2016). Repression of *ubh-4* (*C. elegans* DUB gene) by DAF-16 induces proteasome activity (Matilainen et al., 2013).

Lysosomal proteolytic activity deteriorates with aging (Sarkis et al., 1988; Kaushik et al., 2021). In *C. elegans*, many studies have reported the direct link between autophagy and longevity [e.g. (Melendez et al., 2003; Hansen et al., 2008; Chang et al., 2017), and many more]. Mutational inactivation of autophagy genes (*unc-51, bec-1, atg-18, atg-9, lgg-1*) shortens *C. elegans* lifespan (Toth et al., 2008). Many autophagy genes contribute to longevity paradigms; *Bec-1, unc-51, lgg-1,* and *atg-18* are crucial for lifespan extension through the ILS, TOR signaling, or under conditions of DR (Melendez et al., 2003; Jia and Levine 2007; Hansen et al., 2008; Kenyon 2010). Activation of the AMPK pathway to promote longevity is also autophagy-dependent (Egan et al., 2011; Mihaylova and Shaw 2011). Furthermore, some autophagy receptors promote autophagy-dependent proteostasis and longevity in a tissue specific manner (Kumsta et al., 2017).

The transcriptional regulation of autophagy genes affects longevity and some transcription factors extend lifespan or delay aging in an autophagy-dependent manner in *C. elegans* (Lapierre et al., 2015; Minnerly et al., 2017; Liu et al., 2020; Kaushik et al., 2021). For example, HLH-30/TFEB regulates multiple autophagy genes (*atg-18, vha-16, lmp-1, lipl-1, lipl-3*) and is required for lifespan extension in *glp-1, eat-2, daf-2, clk-1, rsks-1,* and TOR mutants, indicating roles in multiple longevity paradigms (Lapierre et al., 2013). HLH-30 is also implicated in lipid metabolism to promote longevity (Lapierre et al., 2011). PHA-4 regulates autophagy gene expression and is required to extend lifespan (Panowski et al., 2007; Sheaffer et al., 2008; Zhong et al., 2010; Lapierre et al., 2011). DAF-16 also regulates autophagy gene expression to extend longevity (Wang et al., 2008; McColl et al., 2010; Lapierre et al., 2015; Kaushik et al., 2021). The Myc-Mondo:Mlx transcriptional activation complex and the Mad:Max transcriptional repression complex links autophagy to longevity through ILS, DR, and germline signaling (Johnson et al., 2014; Nakamura et al., 2016). The homeodomain interacting protein kinase (*hpk-1*) is a transcriptional co-factor and nuclear kinase that regulates longevity and preserves proteostasis, at least in part, through an essential role in the induction of autophagy in response to inhibition of TOR or under conditions of DR (Das et al., 2017). How these transcription factors regulate autophagy under stress conditions (i.e., diverse metabolic or non-metabolic stressors), across cell types (either cell-intrinsically or cell non-autonomously), or compensate and coordinate specific types of autophagy, are all areas for future investigation.

### Transcriptional Regulation of the Proteostatic Network

Vital components of the PN are adaptive transcriptional responses activated in response to acute or chronic damage to the proteome, as well as in response to metabolic and mitogenic signals. The most well-characterized adaptive transcriptional responses to proteotoxic stress are the HSR, OSR, mitochondrial unfolded protein response (mitoUPR), and ER-UPR. The HSR, ER-UPR, and mitoUPR are induced in response to proteotoxic stress within the cytosol/nucleus, ER, and mitochondria, respectively. Regulation of these adaptive transcriptional programs are critical aspects of the larger PN that act in concordance with each other and additional protein quality control components. There is growing evidence for crosstalk and compensatory mechanisms among these adaptive responses. Furthermore, each has cell-intrinsic and non-autonomous components [reviewed in (Dillin et al., 2014; Taylor et al., 2014; van Oosten-Hawle and Morimoto 2014; Morimoto 2019)]. Below, we focus on HSF1, which maintains proper function of the cytoplasmic and nuclear proteomes. Breakdown of the OSR, mitoUPR, and ER-UPR components of the PN have direct ties to cancer, neurodegenerative disease, and aging, but are beyond the scope of this review [see (Ryan and Hoogenraad 2007; Sykiotis et al., 2011; Wolff and Dillin 2013; Dufey et al., 2014; Jovaisaite et al., 2014; Mottis et al., 2014; Wolff et al., 2014; Karagoz et al., 2019a; Karagoz et al., 2019b; Preissler and Ron 2019) for detailed reviews on the OSR, mitoUPR and ER-UPR]. For a comprehensive review of transcriptional and epigenetic regulation of stress response in *C. elegans* longevity, see (Denzel et al., 2019).

### Tissue-Specific Utilization of the PN

While core components of the chaperone system are uniformly expressed across tissues, each cell type preferentially utilizes specific subsets of molecular chaperones, presumably in alignment with the demands of a proteome that is unique to each particular cell type [(Shemesh et al., 2021), and reviewed in (Labbadia and Morimoto 2015a; Sala et al., 2017)]. For example, the proteomes of cells within the pancreas and muscle significantly differ; the former have elevated expression of secreted proteins, while the latter are enriched in mitochondrial localized proteins (compared to the mean expression in all tissues) (Sala et al., 2017). Thus, it is not surprising that ER-specific chaperones are the major class of chaperones expressed in the secretory tissues of the pancreas, small intestine, and liver. In contrast, sHSPs are overrepresented in skeletal and cardiac muscle, consistent with their role in maintaining the folding of filament components. In accordance with their key role in proteome maintenance, the proportion of HSP70, HSP40, and HSP90 chaperones are relatively constant across tissues (Taipale et al., 2010; Guisbert et al., 2013; Sala et al., 2017; Nisaa and Ben-Zvi 2021). Nevertheless, specific members within these families, as well as other PN components, can be enriched to support specialized functions within a given tissue (Hageman and Kampinga 2009). Consistently, the regulatory pathways that control the heat shock response (HSR) comprise a heat shock regulatory network with tissue-selective effects: in total 59 regulators of the HSR were identified through a genome-wide functional genomic screen and include both molecular chaperones and additional components of the PN (Guisbert et al., 2013). Recent analysis of the transcriptional landscape of molecular chaperones have delineated core chaperones expressed across human tissues from variable chaperones differentially expressed to match tissue specific requirements, which collectively form conserved tissue-specific functional networks (Brehme et al., 2014; Shemesh et al., 2021). Interestingly, these networks are formed during development and differentiation to rewire the cell chaperoning capacities and alter usage of mechanisms to maintain protein quality control (Nisaa and Ben-Zvi 2021; Shemesh et al., 2021). Thus, when considering targeting the PN for the effective treatment of disease, one must account for the tissue of origin, the unique nature of the proteome within that tissue, cell fate/differentiation status, and specialized components of the PN acting within a tissue or cell type.

### Cell Non-Autonomous Regulation of Proteostasis and Longevity are Linked

In addition to cell-intrinsic mechanisms, organisms maintain proteostasis through cell non-autonomous mechanisms. To date, the majority of these discoveries have come from studies in *C. elegans*. For instance, a regulatory component of the HSR has a cell non-autonomous component; the maintenance of proteostasis throughout the organism is controlled by thermosensory neurons in a serotonin-dependent manner (Prahlad et al., 2008; Tatum et al., 2015). In contrast, a second regulatory component from the GABAergic and cholinergic system normally limits muscle cell proteostasis (Garcia et al., 2007; Silva et al., 2013). Furthermore, a moderate increase in cholinergic signaling in the neuromuscular junction triggers calcium influx to the cytosol of muscle cells, activating a downstream signaling cascade leading to the transcriptional activation of HSF-1, and thereby the expression of molecular chaperones (Silva et al., 2013). These findings suggest that upstream neuronal signals regulate proteostatic mechanisms in distal tissues. The GATA transcription factor: PQM-1, functions as a mediator of transcellular chaperone signaling, acting in either a neuron or intestinal-specific route to trigger *hsp-90* in remote tissues to preserve proteostasis and metastable proteins in muscle cells induces systemic stress response across multiple tissues through transcellular chaperone signaling (van Oosten-Hawle and Morimoto 2014; O’Brien et al., 2018; Morimoto 2019)*.* Moreover, the expression of HSP90 within intestinal or neuronal cells is sufficient to suppress protein misfolding in muscle cells (van Oosten-Hawle et al., 2013). Proteostasis and stress resilience in reproductive adult *C. elegans* are also regulated by communication from internally fertilized embryos (Sala et al., 2020).

Cell non-autonomous signals regulates components of the PN and extends longevity in *C. elegans*. For example, specific downregulation of electron transport chain components in the nervous system causes an increase in the mitoUPR in non-neuronal tissues (Durieux et al., 2011). Surprisingly, neuronal downregulation of the respiratory chain complex IV promotes longevity to a similar level as observed when it is downregulated throughout the Soma, and induces mitochondrial chaperones in the intestine. Furthermore, neuronal proteotoxic stress targeting mitochondria elicits a global induction of the mitoUPR through serotonin signaling (Berendzen et al., 2016). Interestingly, cell non-autonomous regulation of mitochondrial stress has also been observed in mammals, where the fibroblast growth factor 21 (FGF21) mediates a signaling event from muscle to peripheral tissue triggered by mitochondrial dysfunction in mice (Kim et al., 2013). Two lines of research indicate that the ER-UPR is cell non-autonomously regulated in *C. elegans*. First, neuronal expression of the spliced and activated form of the ER-UPR transcription factor X box-binding protein 1 (XBP-1) is sufficient to induce the activation of ER-UPR in intestinal cells, which in turn increases stress resistance and extends longevity (Taylor and Dillin 2013). Second, expression of octopamine receptor 1 (OCTR-1) in ASH, ASI, AIY or ADE chemosensory neurons is necessary to inhibit the activation of XBP-1 and the expression of non-canonical ER-UPR genes in distal cells (Urano et al., 2002; Sun et al., 2011). Interestingly, activation of the ER-UPR within distinct neuronal cell types activate unique responses in peripheral tissues (Higuchi-Sanabria et al., 2020). In mammals, ER-UPR interaction between tissues has been proposed as the mechanism of induction of ER stress in tumor cell lines that promotes the activation of the ER-UPR in macrophages, resulting in the production of pro-inflammatory cytokines leading to tumor growth (Mahadevan et al., 2011). Lastly, gonadal stem cell non-autonomous signaling also links proteotoxic stress resistance and longevity: loss of *C. elegans* gonadal stem cells results in increased somatic maintenance through increased proteosomal activity: overexpression of the 19S proteasome subunit *rpn-6* is sufficient to fortify proteostasis and increase lifespan (Vilchez et al., 2012).

## HSF1: Guardian of the Cytosolic and Nuclear Proteome

Heat shock transcription factors are conserved throughout eukaryotes. The *Drosophila melanogaster, C. elegans,* and *Saccharomyces cerevisiae* genomes each encode one heat shock transcription factor, whereas HSF has expanded in vertebrates to encode four paralogs (HSF 1 through 4). Here we focus primarily focus on HSF1 [for more on HSF family members and their discovery, see (Sorger and Pelham 1988; Wiederrecht et al., 1988; Clos et al., 1990; Rabindran et al., 1991; Liu et al., 1997; Nakai 1999; Gomez-Pastor et al., 2018; Roos-Mattjus and Sistonen 2021)].

### Regulation of HSF1

As a transcription factor, HSF1 is regulated extensively by post-translational modifications including acetylation, sumoylation, and phosphorylation (summarized in Table 1). These modifications regulate HSF1 activity at multiple stages, including release from inhibitors, nuclear translocation, homotrimerization, promoter binding, and recruitment of RNA Polymerase II [reviewed in (Gomez-Pastor et al., 2018; Roos-Mattjus and Sistonen 2021)].

**TABLE 1:** HSF1 post-translational modifications.

Modification	Effect	Signal	Enzyme	References
*Inhibitory Signals*				
Ac-K80	loss of DNA binding		p300/SIRT1	Raychaudhuri et al. (2014)
				Westerheide et al. (2009)
Ph-S121	nuclear export	Metabolic	AMPK	Dai et al. (2015)
	Hsp90 interaction	Inflammation	MAPKAPK2	Wang et al. (2006)
Ph-S303	promotes SUMO-K298	Heat		Hietakangas et al. (2003)
	basal transcriptional repression			Kline and Morimoto, (1997)
			GSK3β	Chu et al. (1996)
				Chu et al. (1998)
				He et al. (1998)
	inhibits granules			He et al. (1998)
	cytoplasmic sequestration by 14-3-3		GSK3β	Wang et al. (2003)
	degradation *via* UPS			Kourtis et al. (2015)
Ph-S307	basal repression			Chu et al. (1996)
				Chu et al. (1998)
				He et al. (1998)
				Kline and Morimoto, (1997)
				Knauf et al. (1996)
				Xia and Voellmy, (1997)
	promote P-S303 by GSK3β		MAPK/ERK	Chu et al. (1996)
				Chu et al. (1998)
				Huang et al. (2018)
				Knauf et al. (1996)
				Wang et al. (2003)
	degradation via UPS			Kourtis et al. (2015)
Ph-S363	basal repression		PKC	Chu et al. (1998)
	loss of DNA binding, inhibits granules		JNK	Dai et al. (2000)
Ph-S216	degradation via UPS	Early Mitosis	PLK1	Lee et al. (2008)
Su-K298	limits activation	Heat	UBC9	Anckar et al. (2006)
				Hietakangas et al. (2003)
				Hietakangas et al. (2006)
				Hong et al. (2001)
*Stimulatory Signals*				
Ph-T142	DNA binding, increased transcription	Heat	CK2	Soncin et al. (2003)
Ph-S230	increased transcription	Stress	CaMKII	Holmberg et al. (2001)
Ph-S419	nuclear import	Heat	PLK1	Kim et al. (2005)
Ph-S320	nuclear localization	Heat	PKA	Murshid et al. (2010)
				Zhang et al. (2011)
Ph-S326	increased transcription	Heat		Guettouche et al. (2005)
			MEK	Tang et al. (2015)
			mTOR	Chou et al. (2012)
	increased stability, nuclear import		MEK	Tang et al. (2015)
*Additional modifications*				
Ac-K116, 118, 126, 148, 157, 208, 224, 298		Heat	p 300/SIRT1	Raychaudhuri et al. (2014)
				Westerheide et al. (2009)
Ph-S292, 314, 319, 344, 368, 444				Guettouche et al. (2005)
				Olsen et al. (2006)
				Olsen et al. (2010)
Ph-T323,367,369				Mayya et al. (2009)
				Olsen et al. (2006)
				Olsen et al. (2010)
O-glycosylation				Hamiel et al. (2009)

Under normal conditions, HSF1 exists as an inactive monomer, stabilized by hydrophobic interactions between the heptad repeats in the N- and C-terminal regions (Sorger 1990; Sarge et al., 1993; Orosz et al., 1996; Farkas et al., 1998). One model for HSF1 activation is intrinsic activation; HSF1 effectively acts as a sensor responsive to changing thermodynamic conditions and thereby converts from an inactive monomer to an active trimer. This idea that HSF1 functions as an intrinsic “thermosensor” is consistent with *in vitro* data demonstrating that HSF1 can be activated in response to increasing temperature alone (Hentze et al., 2016). A second “chaperone titration model” posits that under basal conditions HSF1 is sequestered within the cytoplasm by molecular chaperones including HSP70, HSP90, and the chaperonin tailless complex polypeptide 1 (TCP1) ring complex (TRiC) (Abravaya et al., 1992; Baler et al., 1992; Baler et al., 1996; Duina et al., 1998; Shi et al., 1998; Zou et al., 1998; Guo et al., 2001; Akerfelt et al., 2010; Neef et al., 2010; Neef et al., 2011; Neef et al., 2014; Zheng et al., 2016). Upon acute proteotoxic stress, such as heat shock, protein misfolding titrates away chaperones that normally sequester HSF1 as a monomer. Released HSF1 monomers trimerize, translocate to the nucleus, bind to DNA promoters, and upregulate transcription of multiple genes. HSF1 activation induces expression of molecular chaperones, which initiates a negative feedback loop to inactivate HSF1 once stress upon the proteome is resolved (Zheng et al., 2016; Krakowiak et al., 2018). HSF1 has been shown to be regulated by additional means, including intrinsic refolding mechanisms (Mosser et al., 1990; Goodson and Sarge 1995; Xia and Voellmy 1997; Farkas et al., 1998; Zhong et al., 1998; Ahn and Thiele 2003), non-coding RNA (Shamovsky et al., 2006) and *in vivo* through cell non-autonomous signals from thermosensory neurons in *C. elegans* (Clark et al., 2007; Prahlad et al., 2008).

### Conserved HSF-1 Regulation in *C. elegans*

There is a general conservation of mechanisms of HSF-1 regulation between *C. elegans* and mammals, yet the precise molecular mechanisms of control and how, for instance, post-translational modifications and direct interactors of mammalian HSF1, intersect with the wealth of genetic information linking *C. elegans* HSF-1 to longevity and stress signaling remains poorly understood*.* In both mammalian cell culture and *C. elegans,* under basal conditions HSF-1 is transcriptionally inactive due to cytoplasmic sequestration. Upon stress in either system, HSF-1 nuclear localization is governed by phosphorylation (Chiang et al., 2012; Dai et al., 2015; Tang et al., 2015). Similarly, nuclear HSF-1 forms stress granules upon heat stress in both *C. elegans* and mammals (Alastalo et al., 2003; Morton and Lamitina 2013). As in mammals, *C. elegans* HSF-1 transcriptional activation is dependent on the regulation of trimerization (Chiang et al., 2012). At least one direct regulator of HSF1 is conserved between mammals and *C. elegans*: the heat shock binding factor 1 (HSB-1) negatively regulates HSF-1 transcriptional activity (Satyal et al., 1998). Other direct regulators in mammals, such as TOR and AMPK, also show genetic interactions in *C. elegans* (discussed below), but whether direct interactions occur in *C. elegans* has yet to be determined. Knockdown of *hsp-70* also activates HSF-1, implying that the chaperone titration model regulation of HSF1 is conserved (Guisbert et al., 2013).

### Secondary Structure of HSF1

**FIGURE 2:**
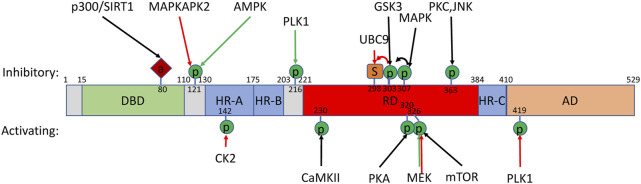
HSF1 post-translational modifications. Schematic of mammalian HSF1 secondary structure with post-translational modifications with known regulators and overall effect on activity. Additional modifications without known regulators are listed in Table 1. Additional regulators where a specific post-translational modification has not been identified have been omitted. “DBD” indicates the helix-turn-helix loop DNA-binding domain. “HR-A/B” and “HR-C″ identify two regions of leucine zippers. “RD” indicates the regulatory domain. “TAD” indicates the transcriptional transactivation domain. Modifications in response to heat (red arrows) or metabolic/mitogenic signals (green) are shown. Amino acid numbers are indicated. “P” indicates phosphorylation. “S” indicates sumoylation. “A” indicates acetylation.

Structurally, HSF1 is highly conserved across metazoan animals (Figure 2). The N-terminal region of approximately the first 100 amino acids is the most well-conserved region of the HSF protein family and encodes a helix-turn-helix loop DNA-binding domain (DBD), which recognizes DNA heat shock elements (HSE) (identified as nGAAn DNA repeats) (Wu 1995). Two regions of leucine zippers (HR-A/B and HR-C) allow oligomerization of HSF1 monomers (Bjork and Sistonen 2010; Anckar and Sistonen 2011; Dayalan Naidu and Dinkova-Kostova 2017). HR-A/B immediately follows the DBD and flanks a regulatory domain (RD) on one side, while HR-C is downstream of the RD. Intrinsic interactions of HR-A/B with HR-C prevents spontaneous HSF1 trimerization and activation under basal conditions. Approximately the last 100 amino acids of HSF1 contains the trans-activation domain (TAD), which is the region through which HSP70 interacts with HSF1.

### HSF1 Regulation Through Phosphorylation

Phosphorylation of mammalian HSF1 is one of the most well studied mechanisms of HSF1 regulation; mass spectrometry and site-directed mutagenesis have identified phosphorylation on multiple serine or threonine residues (S121, S127, T142, S195, S216, S230, S292, S303, S307, S314, S319, S320, T323, S326, S338, S344, S363, T367, S368, T369, S419, and S444) [e.g. (Guettouche et al., 2005; Anckar and Sistonen 2011; Xu et al., 2012; Gomez-Pastor et al., 2018; Roos-Mattjus and Sistonen 2021), summary in Table 1]. Some residues appear to be phosphorylated under basal conditions (Chu et al., 1996; Knauf et al., 1996; Kline and Morimoto 1997; Chu et al., 1998; He et al., 1998) whereas other sites undergo inducible phosphorylation (Holmberg et al., 2002). Stress induces phosphorylation at multiple residues (i.e., hyperphosphorylation) and is concurrent with transactivation (Cotto et al., 1996; Xia and Voellmy 1997; Holmberg et al., 2001; Holmberg et al., 2002; Guettouche et al., 2005).

Most phosphorylation events repress transcriptional activity. For example, sequential phosphorylation of S307 by mitogen-activated protein kinase/extracellular signal-regulated kinase (MAPK/ERK) and S303 by glycogen synthase kinase 3 (GSK3) (Chu et al., 1996; Chu et al., 1998; He et al., 1998) recruits 14-3-3 proteins to export HSF1 from the nucleus and sequester it in the cytoplasm (Wang et al., 2003). Several serine residues are targeted by multiple kinases to repress HSF1 activity: acute inflammatory signals activate mitogen-activated protein kinase 2 (MAPK2) to phosphorylate HSF1 at S121, which promotes direct interaction with HSP90, subsequently reducing binding to DNA at heat shock elements and therefore lessening the activation of corresponding gene expression (Wang et al., 2006). This same residue is also phosphorylated by AMPK under conditions of metabolic stress to impair HSF1 activity by impeding nuclear translocation, thereby rendering cells sensitive to proteotoxic stress (Dai et al., 2015).

HSF1 (S363) phosphorylation also inhibits activity and is the target of both protein kinase C (PKC) and c-Jun N-terminal kinase (JNK). PKC was shown to inhibit HSF1 without affecting trimerization or binding to heat shock elements, and it may limit HSF1 activity during increases in PKC activity, such as after activation by growth receptors (Chu et al., 1998). JNK, a stress-responsive member of the MAPK pathway, also inactivates HSF1 through S363 phosphorylation, which rapidly clears HSF1 from the sites of transcription (Dai et al., 2000). Thus, the outcome of HSF1 (S363) phosphorylation on DNA binding is context-dependent. JNK has also been shown to phosphorylate the TAD of HSF1 after severe heat shock, and JNK inhibitors prevent the activation of HSF1 target genes, but whether this is occurring at S363 is unclear (Park and Liu 2001). Collectively, these findings indicate that diverse negative regulatory signals can converge on a common HSF1 residues to limit activity.

HSF1 phosphorylation can also activate transcription, which has been best-studied in the context of hyperphosphorylation due to acute heat stress. By mass spectrometry, twelve serine residues were identified that are phosphorylated after heat stress (Guettouche et al., 2005). During heat stress, Polo-like kinase (PLK1), one of the major protein kinases involved in cell division, and specifically in APC/C regulation, directly phosphorylates HSF1 on S419, and a S419A mutation inhibits HSF1 translocation to the nucleus, suggesting this is an early step in HSF1 activation (Kim et al., 2005). However, PLK1 also phosphorylates HSF1 (S216) in early mitosis, facilitating degradation, which is essential for proper mitotic progression (Lee et al., 2008). Thus, PLK1 can regulate HSF1 in opposing manners through phosphorylation of different residues in response to distinct signals. Similar to PLK1 action at HSF1 (S419), protein kinase A (PKA) phosphorylates HSF1 (S320) in response to heat shock, and this modification is required for translocation to the nucleus, DNA binding at HSE, and to activate expression of molecular chaperones (Murshid et al., 2010; Zhang et al., 2011). Casein kinase 2 phosphorylates HSF1 on T142 and mutation to alanine (T142A) inhibits trans-activation of *HSP70* by HSF1 and blocks binding to HSE, without affecting translocation to the nucleus (Soncin et al., 2003).

Both S230 and S326 have also been shown to be essential for the increased HSF1 transcriptional activity in response to acute heat shock (Holmberg et al., 2001; Guettouche et al., 2005; Tang et al., 2015). S230 lies within a consensus site for calcium/calmodulin-dependent protein kinase II (CaMKII), and CaMKII overexpression enhances both the level of *in vivo* S230 phosphorylation and transactivation of HSF1 (Holmberg et al., 2001). Furthermore, S230 is not needed for either the heat-induced DNA-binding activity or granule formation but is essential for the transcriptional activity of HSF1 (Holmberg et al., 2001). HSF1 (S326) has been shown to be directly phosphorylated by mTOR, and inhibition of mTOR limits induction of molecular chaperones (Chou et al., 2012), yet it is unclear whether mTOR is essential for HSF1 (S326) phosphorylation after heat stress. mTOR is a major regulator of cellular growth and translation, which suggests that mTOR regulation of HSF1 activity may directly balance the total protein abundance within the proteome and molecular chaperone abundance in response to cell size. Interestingly, MAPK/ERK Kinase, or mitogen-activated protein kinase (MEK), phosphorylates S326 both *in vitro* and *in vivo* after heat stress to activate and stabilize HSF1 to preserve proteostasis (Tang et al., 2015). Furthermore, under heat stress, ERK, MEK, and HSF1 assemble into a ternary protein complex wherein ERK suppresses HSF1 (S326) phosphorylation through inhibitory phosphorylation of MEK.

The emerging picture of regulation of mammalian HSF1 through phosphorylation is one of an integrated, combinatorial process. Indeed, in contrast to the aforementioned studies where HSF1 function was compromised after mutating a single phosphorylation site, an HSF1 isoform with 15 phosphorylation sites mutated, including residues targeted by both inhibitory and stimulatory signals, was still able to translocate to the nucleus, bind DNA, and activate transcription (Budzynski et al., 2015). Thus, HSF1 activity is carefully balanced between positive and negative regulators, which is likely dependent on cell type and context. While in *C. elegans* HSF-1 has been shown to undergo phosphorylation (Chiang et al., 2012), only a fraction of modified amino acids in mammalian HSF1 are directly conserved (Supplementary File S1). Furthermore, the molecular and cellular details of regulation, and subsequent consequences on stress response, proteostasis, and longevity in an intact multicellular organism remains underexplored.

### Ubiquitination of HSF1 and Degradation *via* the UPS

HSF1 protein levels are also regulated via degradation by the UPS. HSF1 can be ubiquitinated and degraded by the Skp1–Cul1–F box (SCF) ubiquitin ligase complex (Skaar et al., 2013). Ubiquitination of HSF1 by the SCF complex occurs during mitosis upon phosphorylation of HSF1 (S216) by PLK1, which releases HSF1 from the Cdc20 complex (Lee et al., 2008). In another study, the SCF complex was shown to target HSF1 for degradation *via* F-box and WD repeat domain containing protein 7 alpha (FBXW7α), a substrate-targeting subunit of the SCF complex. Interestingly, interaction occurs through a conserved degron motif phosphorylated by GSK3β and ERK1; FBXW7α ubiquitylates HSF1, and loss of FBXW7α results in impaired degradation of nuclear HSF1 and defective HSR attenuation (Kourtis et al., 2015). This suggests the possibility that distinct stimuli converge through phospho-regulation of HSF1 to recruit the SCF complex and target HSF1 for degradation. HSF1 may be transported to the proteasome through the Filamin A interacting protein 1-like (FILIP-1L) protein, which has been found in a complex with HSF1, Hsp72, and the ubiquitin-binding domain of hHR23A, a receptor that transports polyubiquitinated proteins to the proteasome for degradation; cells co-expressing HSF1 and FILIP-1L exhibit reduction in the HSF1 protein levels and inhibition of stress granule formation following exposure to heat shock (Hu and Mivechi 2011).

### Sumoylation of HSF1

HSF1 is negatively regulated through sumoylation of HSF1 in both *C. elegans* and mammalian cell culture (Hong et al., 2001; Hietakangas et al., 2003; Anckar et al., 2006; Hietakangas et al., 2006; Das et al., 2017). SUMO protein catalyzes a small ubiquitin like modification, which frequently targets transcription factors to limit activity (Gill 2005; Cubenas-Potts and Matunis 2013; Deyrieux and Wilson 2017; Wotton et al., 2017). Mammals have four genes encoding SUMO, while *C. elegans* possess a single gene, *smo-1* (Kamitani et al., 1998; Jones et al., 2002). In mammals, mild heat stress results in HSF1 (S303) phosphorylation, which promotes subsequent SUMO-1 addition to HSF1 (K298) and limits HSF1 transcriptional activation. More severe and prolonged heat stress results in desumoylation and increased expression of heat shock genes (Hietakangas et al., 2003; Anckar et al., 2006). Thus, sumoylation has been proposed to act as a mechanism to fine-tune HSF1 activity to levels of acute proteotoxic stress (i.e., protein misfolding). Mapping of the human SUMO proteome has identified many additional lysine residues of HSF1 that putatively undergo sumoylation, but the biological significance of these modifications remains unknown (Hendriks et al., 2017; Gomez-Pastor et al., 2018; Roos-Mattjus and Sistonen 2021). In *C. elegans,* sumoylation also limits the inducibility of the HSR (Das et al., 2017), indicating that this mechanism regulates HSF-1 activity *in vivo*, as well as in mammalian cell culture. Consistently, loss of the SUMO isopeptidase *ulp-1* shortens lifespan under conditions of mild heat stress (Samuelson et al., 2007). Additionally, the transcriptional cofactor HPK-1 prevents HSF1 sumoylation under basal conditions (Das et al., 2017). Interestingly the yeast ortholog of HPK-1, Yak1, which directly phosphorylates HSF1 in response to altered metabolic conditions, is induced by heat stress and is required for thermal stress survival (Garrett et al., 1991; Hartley et al., 1994), suggesting that a regulatory role of HPK-1 upon HSF1 is evolutionarily conserved.

### Acetylation of HSF1

HSF1 is also regulated through acetylation. At least nine lysine residues of HSF1 have been shown to be acetylated (Table 1). Perhaps the most well-studied is K80 acetylation of HSF1; the transcriptional co-activator p300/CBP (CREB-binding protein) acetylates HSF1 (K80) to attenuate the HSR by inhibiting DNA binding. This activity is opposed by the deacetylase activity of SIRT1: downregulation of *SIRT1* resulting in the weaker induction of molecular chaperones due to greater acetylation of HSF1, which prevents binding to HSE (Westerheide et al., 2009; Raychaudhuri et al., 2014). Interestingly, acetylation of HSF1 has been found to both increase and decrease stability (Westerheide et al., 2009; Kim et al., 2016); acetylation at different positions alters the HSF1 stability. Specifically, acetylation of HSF1 at position K118 and K80 lead to attenuation of the HSR due to HSF1 degradation by the ubiquitin-proteasome. In contrast, acetylation at position K208 and K298 stabilizes HSF1 (Raychaudhuri et al., 2014). As SIRT1 is a critical deacetylase of HSF1, it is tempting to speculate that SIRT1 may promote HSF1 stability or degradation depending on the position of acetylation.

### Additional Direct Regulators of Mammalian HSF1

A number of additional proteins have been identified that regulate HSF1 activity through direct interactions. For example, heat shock factor binding protein 1 (Hsbp1) is a negative regulator of HSF1 activity (Satyal et al., 1998). CHIP, Ral-binding protein 1 (RalBP1), and Death-associated protein 6 (Daxx) function in the activation of HSF1 (Dai et al., 2003; Hu and Mivechi 2003; Boellmann et al., 2004). Metastasis-associated protein 1 (MTA1) and Cdc20 are additional HSF1 regulators (Khaleque et al., 2008; Lee et al., 2008).

## Control of Gene Expression by HSF1

HSF1 preserves proteome vitality by commanding a transcriptional program whose physiological purpose is to maintain proper folding and function of the proteome in the face of both global and localized forms of protein misfolding stress. This transcriptional program marshals multiple chaperone systems when protein homeostasis is compromised, either in response to intrinsic or extrinsic cues. In addition to regulating the HSR, HSF1 activity is also responsive to metabolic and mitogenic signals and plays an important role in development and organismal longevity [reviewed in (Li et al., 2017)]. Additionally, HSF1 participates in physiological and pathological processes including: differentiation, immune response, multidrug resistance, longevity, neurodegeneration, and cancer. Interestingly, recent study comprehensively cataloged all known HSF1 target genes and preformed an enrichment analysis of HSF1 targets across tissues, cell types, and organisms (hsf1base.org) (Kovacs et al., 2019) and found that HSF1 targets, expressed in all tissues and cell types, are generally related to maintaining proteostasis. Furthermore, HSF1 targets that are conserved across various animal taxa operate mostly in cellular stress pathways (e.g., autophagy), chromatin remodeling, ribosome biogenesis, and aging; highlighting the diverse roles for HSF1 in regulating gene expression.

### The Heat Shock Response (Unfolded Protein Response in the Cytosol and Nucleus)

The “heat shock response,” defined as the rapid induction of heat shock proteins, was initially described in *Drosophila* 60 years ago (Ritossa 1962). We now know that the HSR is an ancient genetic program shared across all organisms and constitutes one key component of a larger network that responses to stress on the proteome. The HSR could more accurately be described as the Unfolded Protein Response to proteotoxic stress within the cytosol and nucleus, analogous to the mitoUPR and ER-UPR.

Protein stability, and therefore normal cellular function, are highly sensitive to changes in temperature. Acute heat stress not only denatures and aggregates proteins, but also damages the cytoskeleton, breaks down organelles such as Golgi apparatus and the ER, diminished the numbers of functional mitochondria and lysosomes, and produces cytoplasmic stress granules. The cellular consequences of this damage are: a collapse of actin and microtubule networks, disruption of intracellular transport, decreased availability of ATP, a global decrease in translation, a drop in cytosolic pH, and cell cycle arrest [reviewed in (Richter et al., 2010)]. Due to the inherent danger heat has on cellular function, it is not surprising that transcriptional programs evolved early in evolution to respond to heat. Life exists in a wide range of temperatures, for example *Pyrodictium abyssi* grows in hot vents over 100°C, and species of the *Thermoproteus* genus live in boiling mud. Yet, shifting *Pyrodictium occultum* from 102°C to 108°C induces transcriptional changes in response to heat stress (Stetter 2006). Thus, organisms thrive in only a narrow temperature range, and shifts of only a few degrees induces a universal and ancient transcriptional response to heat: the “heat shock response” (Brown and Lupas 1998; Takai et al., 1998; D'Amico et al., 2006; Richter et al., 2010).

Previous transcriptional and proteomic studies have identified a vast number of heat-inducible genes, which are involved in diverse cellular processes. The HSR not only induces the expression of molecular chaperones, but also: increases protein degradation *via* autophagy and expression of proteasome subunits, promotes stabilization of cellular energetics *via* altered expression of metabolic enzymes, inhibits unnecessary processes through the activation of additional regulatory proteins, induces repair of DNA/RNA and changes in gene expression to sustain cellular structures, and repairs membranes to restore transport and detoxification within the cell [reviewed in (Richter et al., 2010)].

HSF1 also restores proteostasis after stress by increased expression of genes involved in autophagy. As previously mentioned, autophagy is a crucial protagonist of the proteostasis network that functions to recycle cytosolic components after stress, including: toxic protein aggregates, nutrient deprivation, hypoxia, and damaged organelles, among others (Kroemer et al., 2010). In *C. elegans*, heat shock and *hsf-1* overexpression induce autophagy in multiple tissues (Kumsta et al., 2017). In mammals, HSF1 regulates the phosphorylation and activity of the SQSTM1/p62 autophagy receptor, suggesting that the HSF1 stress response pathway is involved in autophagic clearance of protein aggregates (Watanabe et al., 2017). Moreover, HSF1 controls autophagy activity induced by chemotherapeutic agents by regulating the transcription of autophagy-related protein 7 (ATG7) (Desai et al., 2013).

### The Role of HSF1 in Development is Distinct From the HSR

Periods of rapid growth during development require proteome expansion, in turn demanding an expansion of the PN to regulate developmental processes. Certain windows during development demand an excess amount of energy and nutritional resources. Organisms consequently experience stress during stages where meeting these needs requires divergence from optimal developmental trajectory (Puscheck et al., 2015). In response to stress, HSF1, along with mammalian paralogs HSF2 and HSF3, acts to compensate and ensure survival of the developing organism (Akerfelt et al., 2010; Puscheck et al., 2015). The role of HSF1 in development with and without canonical heat shock stressors is still under ongoing investigation in other model systems.

In *Drosophila,* mutations in a single base of the HSF coding sequence causes arrest at the first or second larval instar stage of development. However, HSF mutations induced past these larval stages do not affect cell growth or viability under normal conditions, suggesting that HSF is only required in the early stages of development in *Drosophila* (Table 2). Additionally, the expression of canonical heat shock genes did not change in these mutants, suggesting that the developmental role of HSF1 could be distinct from the HSR (Jedlicka et al., 1997).

**TABLE 2:** HSF1 loss and organismal development.

Mutation/allele	Outcome	References
*Drosophila melanogaster*		
Q78TAA	lethal at 1st or 2nd instar stage of development; homozygous lethal	Jedlicka et al. (1997)
Q373TAG	lethal at 1st or 2nd instar stage of development; decreased viability of adults	
S99N (DNA binding domain)	lethal at 1st or 2nd instar stage of development; homozygous lethal	
V57M (DNA binding domain)	lethal at 1st or 2nd instar stage of development; temperature sensitive	
*Caenorhabditis elegans*		
sy441 (truncation, lacks transactivation domain)	egg laying defect, arrested at the L2–L3 stage above 15°C	Hajdu-Cronin et al. (2004)
*ok600* (frameshift deletion, putative null)	L2–L3 stage arrest	Li et al. (2016)
*rsks-1(0); hsf-1(sy441)*	rescued developmental phenotype	Chisnell et al. (2018)

The developmental transcriptional program of HSF-1 in *C. elegans* is distinct from the canonical HSR (Li et al., 2016; Li et al., 2017). *hsf-1* null mutants arrest at the L2-L3 larval stage of development (Table 2). Activation of HSF-1 during development depends on a GC-rich E2F/DP transcription factor binding to a motif that allows HSF-1 to bind to a heat shock element distinct from the classical HSR. Through this, E2F and HSF-1 facilitate regulation of biogenesis and anabolic metabolism during development. However, loss of *hsf-1* also results in lower basal levels of molecular chaperones (Chiang et al., 2012). Additionally, knockdown of components of the mTOR pathway can rescue these defective developmental phenotypes. Specifically, rescue utilizing either knockdown of the TORC1 component *daf-15* (Raptor), a positive regulator of mTORC1 *ragc-1* (orthologous to RAG GTPase), or loss of the downstream effector that regulates rates of translation; *rsks-1* (ortholog of S6 Kinase), prevented developmental arrest (Chisnell et al., 2018). This implies that decreased rates of protein synthesis resulting from mTORC1 inactivation mitigates damage to the proteome associated with loss of HSF function. Alternatively, decreased mTORC1 activity may rescue *hsf-1* developmental defects resulting from diminished basal chaperone expression, at least in part by reducing either the total concentration or specific components of the cellular proteome.

## HSF-1 Integrates Diverse Metabolic and Stress Signals to Preserve Proteostasis and Longevity

### HSF-1 Preserves Longevity

HSF-1 has emerged as a key regulator of organismal longevity through the integration of signals of cellular energy metabolism and diverse forms of stress. This has been well studied in *C. elegans;* loss of *hsf-1* shortens lifespan, impairs survival to a diverse array of cellular stresses, and compromises proteostasis. Conversely, *hsf-1* overexpression increases lifespan, stress resistance, and delays age-associated proteostatic decline (Garigan et al., 2002; Hsu et al., 2003; Morley and Morimoto 2004; Kourtis et al., 2012; Das et al., 2017). Recently, it has been shown that HSF-1 requires the transcriptional cofactor *hpk-1* to extend longevity, to induce molecular chaperones after thermal stress, to enhance hormetic extension of longevity, and is required in conjunction with HSF-1 for maintenance of proteostasis (Das et al., 2017). HPK-1 antagonizes sumoylation of HSF-1 and inhibiting sumoylation increases the induction of molecular chaperones after heat shock (Das et al., 2017). While persistent heat stress is detrimental to nematode survival, either intermittent heat shock or mild hormetic heat shock also extends longevity *via* HSF-1 activation (Das et al., 2017; Kumsta et al., 2017). It is generally believed that hormesis extends longevity by bolstering organismal and cellular stress response pathways, which subsequently offsets aging-related decline in these pathways (Epel and Lithgow 2014).

How HSF-1 extends longevity remains an active area of investigation. Early work suggested that HSF-1 delays aging through expression of molecular chaperones, as overexpression of molecular chaperones can suppress polyglutamine aggregation in body wall muscle and increase *C. elegans* lifespan (Satyal et al., 2000; Hsu et al., 2003; Walker and Lithgow 2003). However, emerging evidence suggesting a more complex picture. First, there are conflicting reports as to whether a hypomorphic *hsf-1* allele (premature stop codon removing the transactivation domain) sensitizes *C. elegans* to heat stress, despite having an impaired HSR (Prahlad et al., 2008; McColl et al., 2010). Interestingly, overexpression of a mutant HSF-1 lacking the transactivation domain is able to increase thermotolerance and lifespan through maintaining cytoskeletal integrity, despite being impaired in the ability to induce molecular chaperones after heat shock (Baird et al., 2014), but it is possible that the truncated *hsf-1* isoform is a neomorph. Another possibility is that HSF-1 may extend longevity through links to other components of the PN, such as the regulation of autophagy (Kumsta et al., 2017). As previously mentioned, efforts to directly link the genetic interactions and cell biological activity of HSF-1 in *C. elegans* to specific HSF1 post-translational modifications that occur in mammalian cells is still lacking, yet it is widely postulated that HSF-1 functions that extend longevity will be the same as those that preserve proteostasis, and by extension possibly delay aging and prevent the manifestation of neurodegenerative age-associated proteotoxic diseases in humans.

### HSF-1 Functions Cell Non-Autonomously to Regulate Proteostasis, Stress Response and Longevity

One transformative discovery in *C. elegans* was the finding that HSF-1 functions cell non-autonomously within neurons to increase longevity and maintain proteostasis in distal tissues (Morley and Morimoto 2004; Prahlad et al., 2008; Prahlad and Morimoto 2011; Douglas et al., 2015), and that this occurs through serotonin signaling (Tatum et al., 2015). Increased neuronal expression of HSF-1 is sufficient to extend longevity and improve stress resistance. Interestingly, signals that increase the HSR in peripheral tissues through thermosensory neuronal circuits are separable from those that increase longevity (Douglas et al., 2015). In accordance to these findings, another study identifies that integrin-linked kinase (ILK) inhibition activates HSF-1 cell non-autonomous effect on stress resistance and lifespan in a thermosensory-dependent manner (Kumsta et al., 2014). Of note, HSF-1 acts in multiple tissues to regulate longevity (Morley and Morimoto 2004) and also acts cell non-autonomously outside of the nervous system. For example, intestinal HSF-1 activity upregulates the *mir-*83/miR-29 secreted microRNA to disrupt macroautophagy both within intestinal and body wall muscle (Zhou et al., 2019). However somewhat paradoxically, hormetic heat shock to activate HSF-1 or HSF-1 overexpression induces autophagy in multiple tissues (Kumsta et al., 2017), yet whether this occurs cell intrinsically or non-autonomously was not explored. Altogether, this implies that strategies to target HSF-1 in the treatment of disease should consider both cell intrinsic changes and effects in distal tissues. It will be interesting to learn whether pro-longevity functions of HSF-1 that are independent of molecular chaperone induction, can improve neuronal proteostasis but not protect cancer cells from chronic proteotoxic stress.

### HSF-1 Integrates Metabolic Signals to Extend *C. elegans* Longevity

The most potent influencers of *C. elegans* longevity sense changes in metabolic status, which in turn leads to the activation of cytoprotective stress response and adaptive transcriptional programs, including the PN. HSF-1 is essential for many of these metabolic pathways or signals to extend longevity: including decreased ILS (Hsu et al., 2003; Morley and Morimoto 2004), germline deficiency (Hansen et al., 2005), reduced TORC1 signaling or inhibition of *rsks-1* (Seo et al., 2013), and dietary deprivation (a distinct method of *C. elegans* DR) (Steinkraus et al., 2008). Furthermore, HSF-1 is subjected to complex regulation at times of simultaneously applying thermal stress and DR, through the integrin-linked kinase PAT-4 (human integrin linked kinase) and the deacetylase SIR-2.1 (Raynes et al., 2012; Kumsta et al., 2014).

Not all longevity signals are dependent on HSF-1, or at least the interrelationship between longevity signaling and HSF-1 are complex. For instance, early work on disruption of the electron transport chain showed increased lifespan independently of *hsf-1* (Hsu et al., 2003). However, more recent work showed lifespan extension of HSB-1/HSF-1 signaling could be in part through modulation of mitochondrial function *via* mediating histone H4-dependent regulation of mtDNA gene expression (Sural et al., 2020), indicating that the complex signaling interactions between the mitochondrial and nuclear genomes is not fully understood. Another example is AMPK and HSF1. In mammals, AMPK activation directly phosphorylates HSF1 to suppress proteotoxic stress response and conversely, either proteotoxic stress or HSF1 itself inactivates AMPK (Dai et al., 2015; Su et al., 2019). In *C. elegans* as AMPK lifespan extension appears to be independent of HSF-1 (Greer and Brunet 2009; Hwang et al., 2014; Kuo et al., 2020). It is unclear whether this regulatory mechanism is simply not conserved, restricted to specific cell types, or compensated for when examined in a multicellular organism.

As discussed above, HSF-1 is required for a specific type of DR (referred to as dietary deprivation) to increase lifespan in *C. elegans* (Hsu et al., 2003; Greer and Brunet 2009). HSF-1 is integral in the extension of longevity under this specific form of DR, but surprisingly not the other methods of DR, indicating that HSF-1 plays a specific role. For example, *eat-2* mutant animals are a genetic model of DR: animals have reduced acetylcholine channel activity, which results in decreased pharyngeal pumping, increased lifespan, and stress resistance. The increased lifespan of *eat-2* animals is *hsf-1* independent (Hsu et al., 2003). In contrast, the increased stress resistance of *eat-2* mutant animals requires *hsf-1* (Shpigel et al., 2019)*.* However, “dietary restriction” is a vague term that attempts to capture all forms of nutritional stress, and multiple methods for inducing DR have been described. Since empirical findings indicate that distinct types of DR have many different genetic requirements, it suggests one of the possible explanations: 1) different feeding regiments or nutrient uptake either titrate a similar signal, and differing genetic requirements are observed when threshold effects trigger a specific response, 2) different types of DR trigger distinct forms of nutrient stress, or 3) some types of DR may trigger multiple forms of nutrient stress, which enact combinatorial responses.

HSF-1 is an essential transcriptional effector of the increased longevity in ILS mutant animals (Hsu et al., 2003; Morley and Morimoto 2004). In wild type animals, DAF-2 inhibits HSF-1 activity through DDL-1 and DDL-2 (Chiang et al., 2012). DDL-1 is homologous to the human coiled-coil domain-containing protein 53 (CCDC53), and DDL-2 is the homolog of human Wiskott-Aldrich syndrome protein and SCAR homolog (WASH2) protein. WASH2 and CCDC53 are both components of the Arp2/3 complex involved in actin polymerization, and intracellular motility of endosomes (Derivery et al., 2009). CCDC53 has also been reported to potentially interact with heat shock factor binding protein 1 (HSBP1) (Rual et al., 2005). Consistently, the Arp2/3 complex as well as multiple components involved in endocytic trafficking to the lysosome are essential for decreased ILS to extend longevity (Samuelson et al., 2007). As noted above, HSF-1 cooperates with DAF-16 through PFD-6, a component of the molecular chaperone prefoldin-like complex, which relays longevity signals from HSF-1 to FOXO under conditions of reduced ILS (Son et al., 2018).

As a key nutrient sensor for NAD + levels, SIRT1 deacetylates HSF1 during nutrient stress promoting stress survival, thus making the acetylation of HSF1 an integral part of sirtuin signaling (Westerheide et al., 2009; Raynes et al., 2013). Deacetylation of mammalian HSF1 frees up its DNA binding domain, allowing it to bind to a HSE, activate the HSR; and inhibition of SIRT1 accelerates the release from the HSE, decreases HSF1 protein expression, and activation of the HSR (Westerheide et al., 2009; Kim et al., 2016). In mammals SIRT1 is also required for maintenance of the proteome as SIRT1 deficiency results in defective protein quality control (Tomita et al., 2015), reinforcing the notion that SIRT1 regulation of HSF1 connects longevity signaling to the maintenance of proteostasis. HSF1 regulation by Sirtuins is conserved in *Saccharomyces cerevisiae,* and *C. elegans* (Anderson et al., 2003; Brunquell et al., 2018). In mammals, AROS (a positive regulator of SIRT1) increases deacetylation of HSF1, while CCAR2 and DBC1 negatively regulate SIRT1 dependent HSF1 deacetylation (Zhao et al., 2008; Raynes et al., 2013). In *C. elegans,* as CCAR-1 negatively regulates SIR-2.1, increasing HSF-1 acetylation and decreasing HSF-1 ability to regulate the HSR (Brunquell et al., 2018), demonstrating a conserved regulatory mechanism for HSF-1 activation. Furthermore, DR induces heat shock gene expression in a *sir-2.1* dependent manner (Raynes et al., 2012). This suggests that signals that modulate Sirtuin function can affect HSF-1. However, increasing NAD + levels promotes longevity in *C. elegans via sir-2.1,* DAF-16 activation, and the mito-UPR, without changing *hsf-1* expression (Mouchiroud et al., 2013). We could not find direct experimental evidence in *C. elegans* to demonstrate that the extended longevity conferred by *sir-2.1* overexpression is *hsf-1* dependent.

Mammalian HSF1 is directly activated by TOR [see above and reviewed in (Antikainen et al., 2017; Saxton and Sabatini 2017; Blackwell et al., 2019)]. Furthermore, experiments in yeast suggest that activated HSF1 might inhibit rapamycin resistance and TOR signaling (Bandhakavi et al., 2008). While a bulk of HSF1 and TOR interactions have been studied in cell culture and yeast, studies in *C. elegans* reveal that inactivation of TOR signaling increases lifespan in a manner dependent on *hsf-1.* Consistently, HSF-1 can also be activated with rapamycin treatment (Seo et al., 2013).

Loss of *C. elegans* germ cells cause a metabolic shift fat mobilization and lipolysis (Wang et al., 2008). This shift in results in aged animals assuming a younger metabolic transcriptional state (Wan et al., 2017). Gonadal stem cells are depleted in *glp-1* mutant animals, which encodes the *C. elegans* ortholog of the NOTCH receptor, and *glp-1* mutations increase lifespan dependent on *hsf-1* (Hansen et al., 2005). Interestingly, shortly after the onset of reproduction the inducibility of the HRS drops dramatically (Labbadia and Morimoto 2015b), and HSF-1 forms nuclear stress bodies at the initiation of reproduction throughout the germline and upon transition to adulthood HSF-1 stress bodies form in most somatic cells (Deonarine et al., 2021). However, this repression of the HSR does not occur in *glp-1* mutant animals (Shemesh et al., 2013) and genetic loss of the germline suppressed nuclear stress body formation with age (Deonarine et al., 2021), which suggests alterations in gonadal stem cell signaling cell non-autonomously reprograms somatic maintenance, at least in part through HSF-1 activity. Interestingly, genetic perturbation of the extracellular vitelline layer of an internally fertilized embryo initiates a transcellular signal to improve proteostasis and stress resistance in an HSF-1 dependent manner (Sala et al., 2020). Thus, HSF-1 links metabolic reprograming and stress signals from the germline to improved proteostasis in the Soma and extend longevity.

## Future Perspectives

Science is an iterative process, where discoveries in one model system informs and generates new hypotheses best tested in others. Emerging technologies, such as clustered regularly interspaced short palindromic repeats (CRISPR) and single-cell RNA sequencing, along with the development of large datasets (e.g., human sequence data), and the field of systems biology hold the promise of translating discoveries made in model organisms to ultimately improve human healthspan. Conversely, model organisms provide a powerful platform to segregate causative mutations from correlations identified within human sequence variation; and can quickly elucidate gene function within an intact metazoan. The proteostasis field is currently undergoing a transformation thanks to the development of AlphaFold, an artificial intelligence program that can accurately predict protein structure (Jumper et al., 2021), which will ultimately provide a predictive iterative framework to merge findings in genetics, structural biology, and biochemistry. These are just a few examples of how advancing technical innovation in the biological sciences will be applied to further unify our understanding of how aging negatively impacts biological systems.

Work in *C. elegans* has been instrumental in understanding the basis of aging, and is well positioned to remain a premiere system of discovery for years to come. For example, in the specific area of transcriptional control of proteostasis, HSF1 regulation through multiple post-translational modifications and direct interactors, reveals the underlying complexity required to maintain proteome function, yet surprisingly little is known about the specific HSF-1 residues that modified within an intact multicellular organism, such as *C. elegans*. Conversely, whether the longevity signals that converge on HSF-1 to delay *C. elegans* aging have analogous levels of regulation in mammals remains an area of exploration. Discovering how HSF-1 is regulated in a cell-type specific manner within an intact multicellular organism would provide insight into how extrinsic and intrinsic signals are integrated *via* HSF-1 to regulate the PN, and how this system breaks down during normal aging. As an integration point for diverse signals of metabolic, mitogenic, and proteotoxic stress; distinguishing between modifications acting as molecular switches, versus providing rheostat regulation to fine-tune gene expression levels, those responsible for selective gene expression and tissue specific regulation, could be paramount in guiding the development of strategies to extend human healthspan.

The past 30 years of aging research has led to a wealth of discovery into the major regulatory mechanisms that have a deterministic role on organismal aging, the molecular and cellular hallmarks of aging, and how organisms respond to myriad forms of stress. Major challenges will be to elucidate how organisms, as biological systems, integrate signals and coordinate adaptive responses to maintain homeostasis, how these processes breakdown during aging, and whether greater understanding will facilitate the development of strategies to maximize human health span and minimize the onset and progression of age-associated diseases.
