# Supplementary material for: HSF-1: Guardian of the Proteome Through Integration of Longevity Signals to the Proteostatic Network
Source: Front Aging. 2022 Jul 8;3:861686. doi: 10.3389/fragi.2022.861686 (PMC9304931; doi:10.3389/fragi.2022.861686)
Supplement: Supplementary file 1 [file Presentation1.PPTX]

## Slide 1
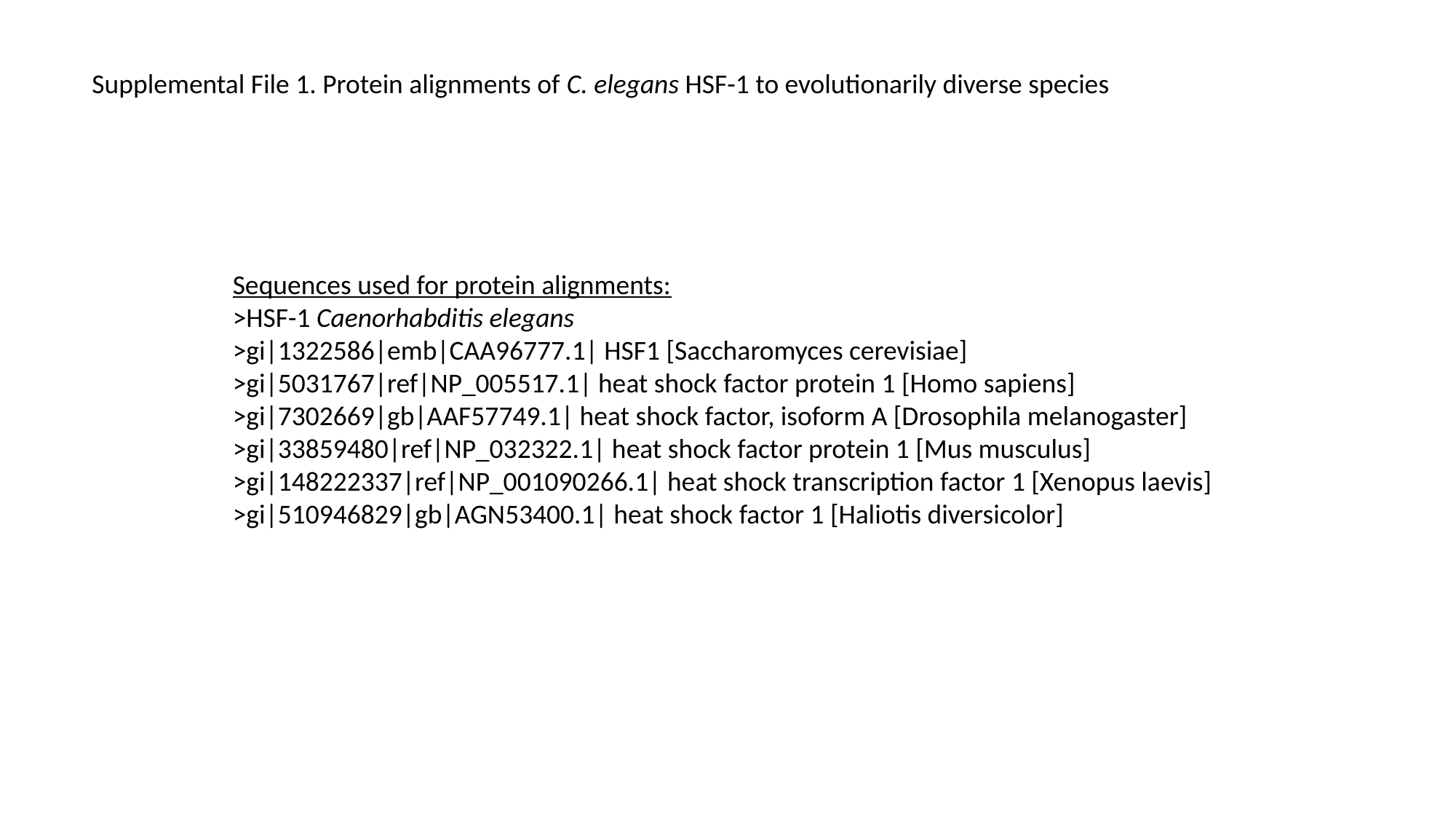

Supplemental File 1. Protein alignments of C. elegans HSF-1 to evolutionarily diverse species
Sequences used for protein alignments:
>HSF-1 Caenorhabditis elegans
>gi|1322586|emb|CAA96777.1| HSF1 [Saccharomyces cerevisiae]
>gi|5031767|ref|NP_005517.1| heat shock factor protein 1 [Homo sapiens]
>gi|7302669|gb|AAF57749.1| heat shock factor, isoform A [Drosophila melanogaster]
>gi|33859480|ref|NP_032322.1| heat shock factor protein 1 [Mus musculus]
>gi|148222337|ref|NP_001090266.1| heat shock transcription factor 1 [Xenopus laevis]
>gi|510946829|gb|AGN53400.1| heat shock factor 1 [Haliotis diversicolor]

## Slide 2
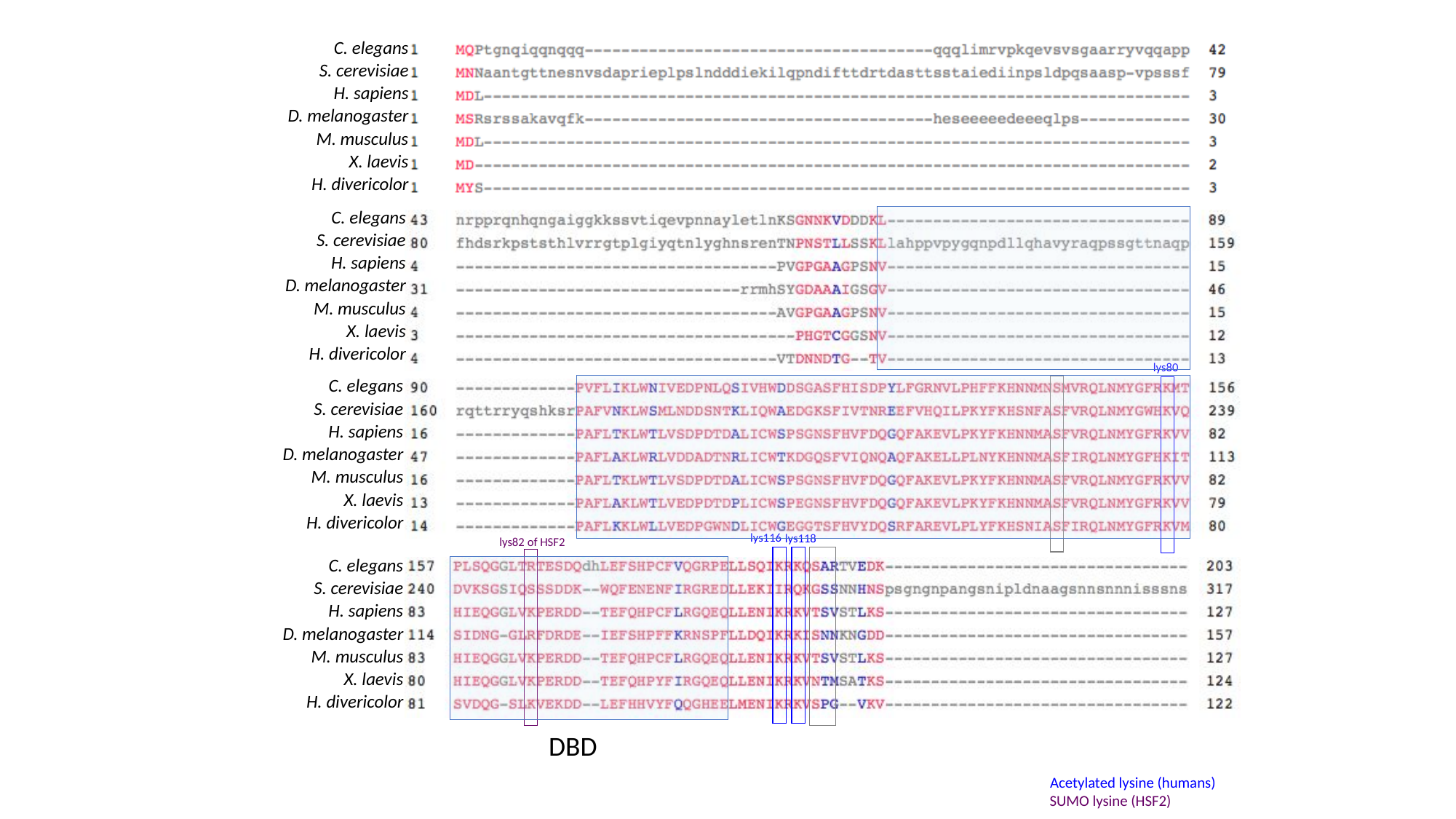

C. elegans
S. cerevisiae
H. sapiens
D. melanogaster
M. musculus
X. laevis
H. divericolor
C. elegans
S. cerevisiae
H. sapiens
D. melanogaster
M. musculus
X. laevis
H. divericolor
lys80
C. elegans
S. cerevisiae
H. sapiens
D. melanogaster
M. musculus
X. laevis
H. divericolor
lys116
lys118
lys82 of HSF2
C. elegans
S. cerevisiae
H. sapiens
D. melanogaster
M. musculus
X. laevis
H. divericolor
DBD
Acetylated lysine (humans)
SUMO lysine (HSF2)

## Slide 3
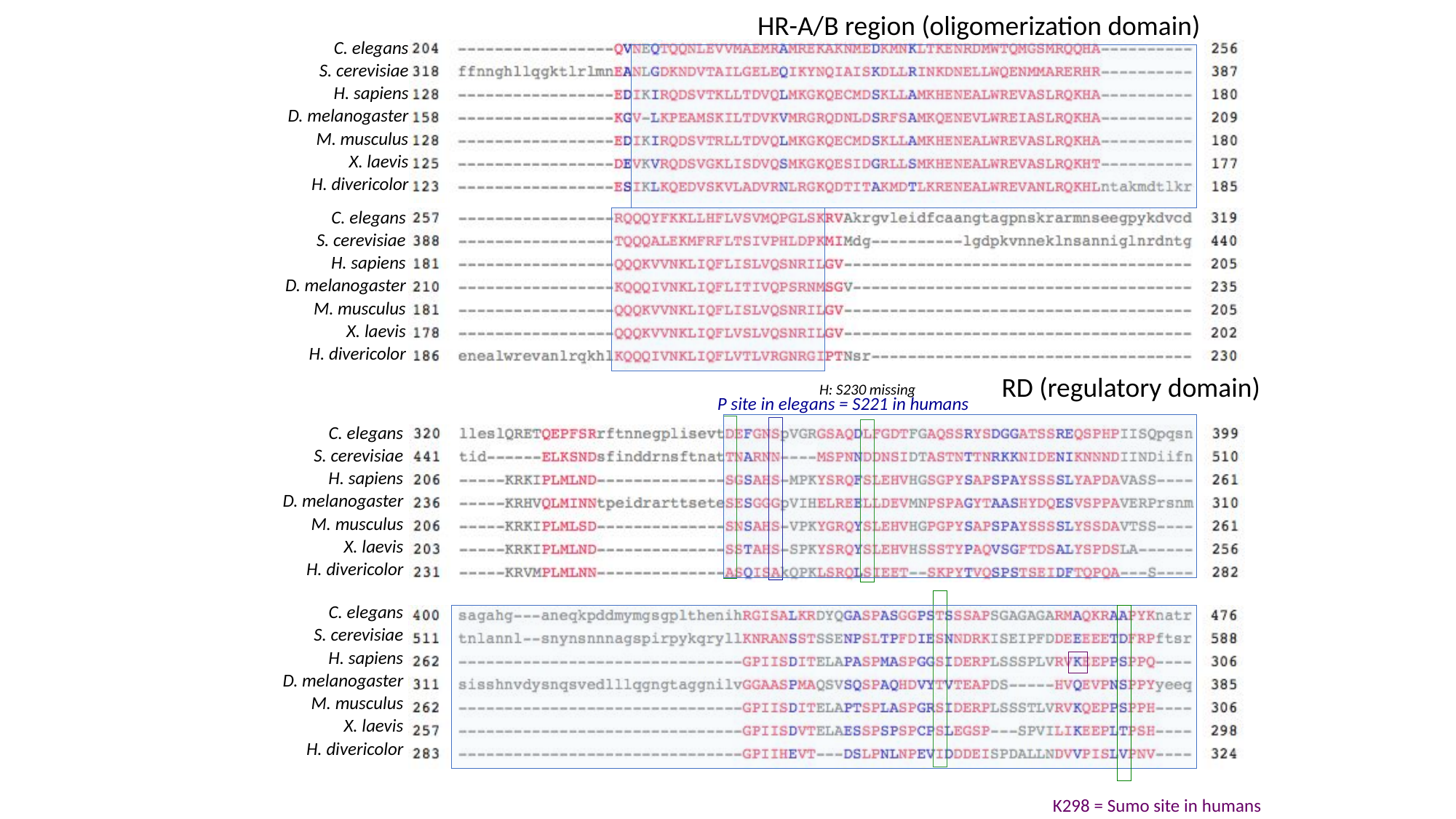

HR-A/B region (oligomerization domain)
C. elegans
S. cerevisiae
H. sapiens
D. melanogaster
M. musculus
X. laevis
H. divericolor
C. elegans
S. cerevisiae
H. sapiens
D. melanogaster
M. musculus
X. laevis
H. divericolor
RD (regulatory domain)
H: S230 missing
P site in elegans = S221 in humans
C. elegans
S. cerevisiae
H. sapiens
D. melanogaster
M. musculus
X. laevis
H. divericolor
C. elegans
S. cerevisiae
H. sapiens
D. melanogaster
M. musculus
X. laevis
H. divericolor
K298 = Sumo site in humans

## Slide 4
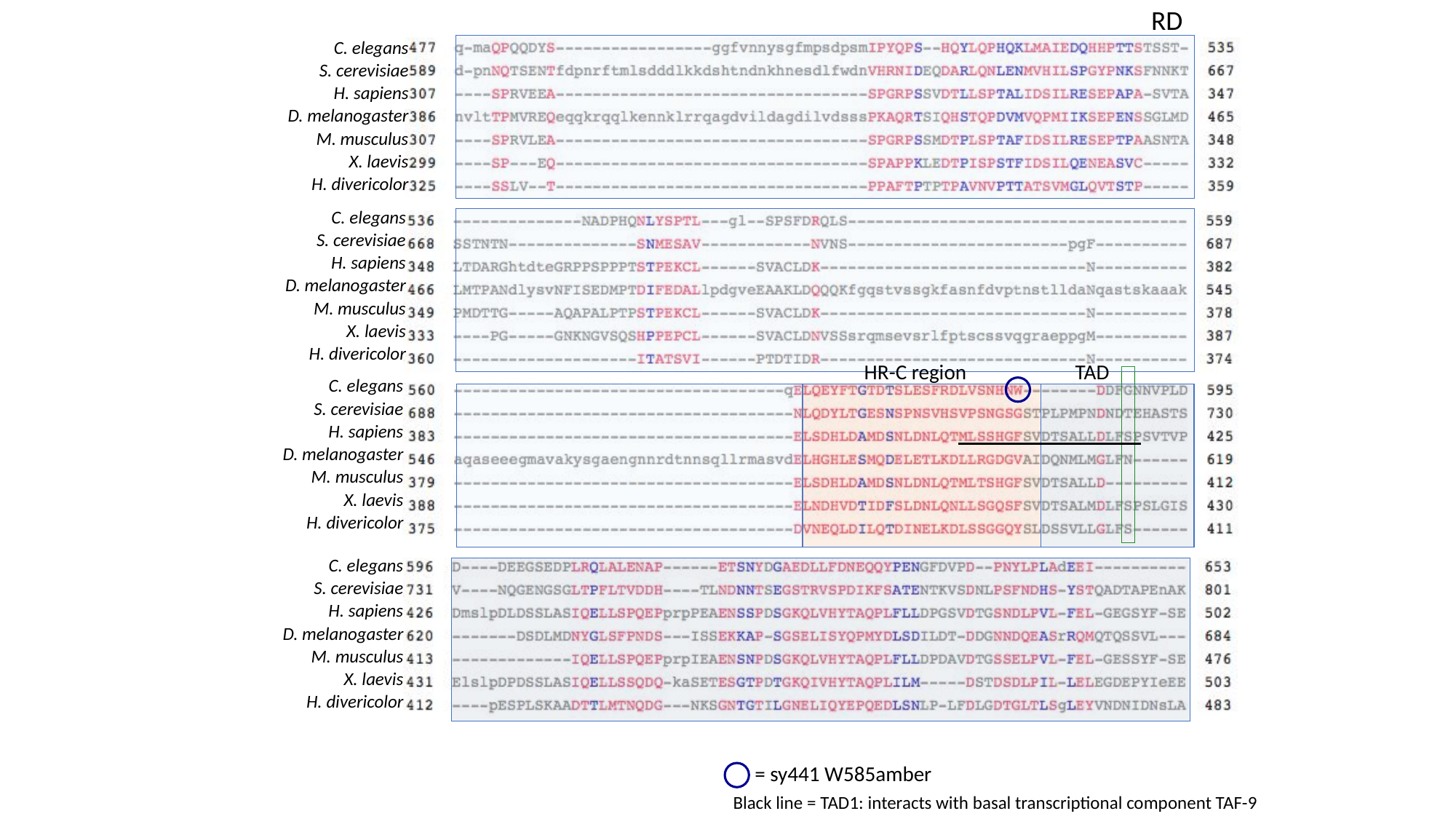

RD
C. elegans
S. cerevisiae
H. sapiens
D. melanogaster
M. musculus
X. laevis
H. divericolor
C. elegans
S. cerevisiae
H. sapiens
D. melanogaster
M. musculus
X. laevis
H. divericolor
HR-C region
TAD
C. elegans
S. cerevisiae
H. sapiens
D. melanogaster
M. musculus
X. laevis
H. divericolor
C. elegans
S. cerevisiae
H. sapiens
D. melanogaster
M. musculus
X. laevis
H. divericolor
= sy441 W585amber
 Black line = TAD1: interacts with basal transcriptional component TAF-9

## Slide 5
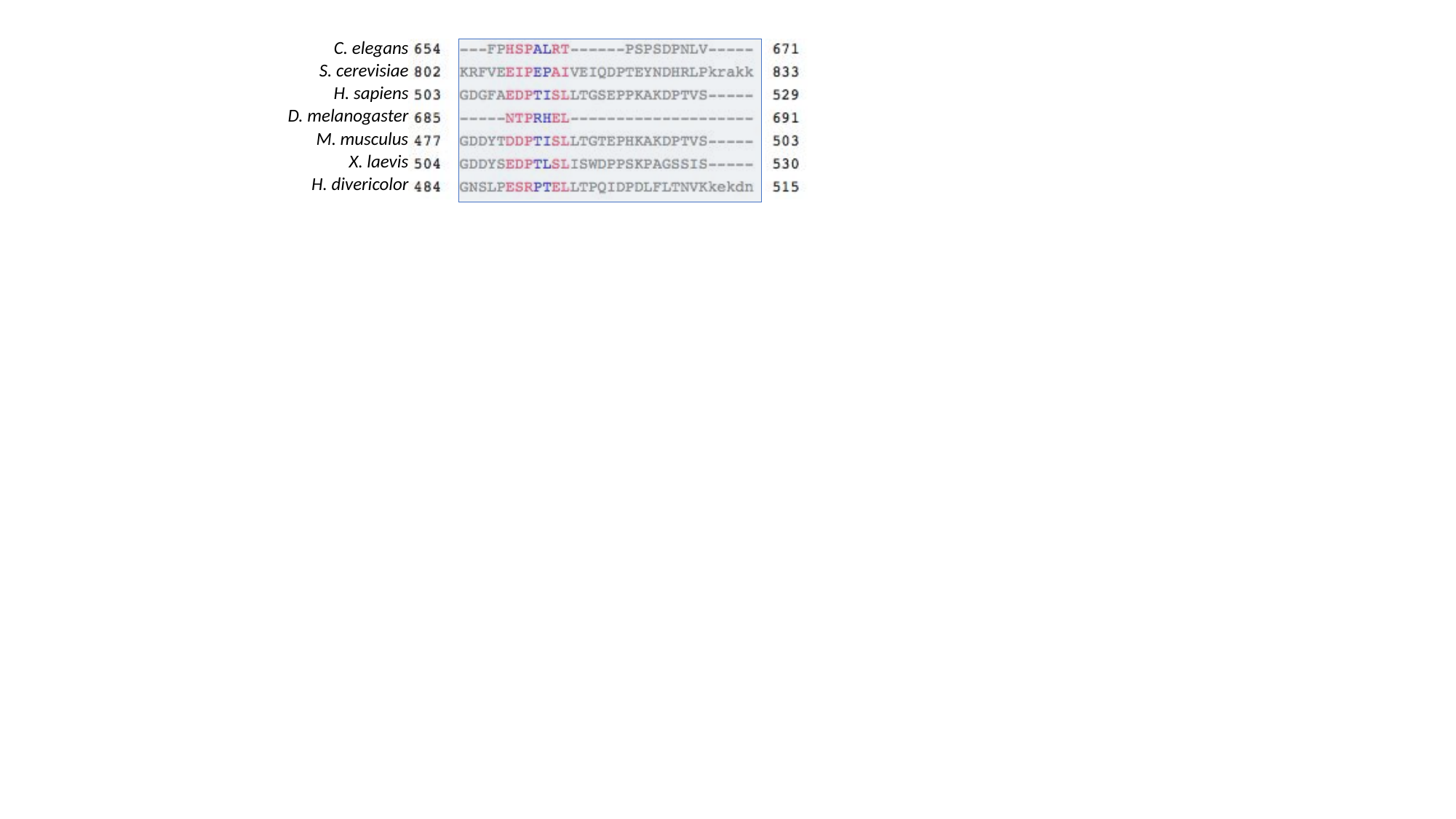

C. elegans
S. cerevisiae
H. sapiens
D. melanogaster
M. musculus
X. laevis
H. divericolor
